# Pericardial Fluid Annexin A1 Is a Marker of Atrial Fibrillation in Aortic Stenosis: A Proteomics Analysis

**DOI:** 10.3390/jpm12020264

**Published:** 2022-02-11

**Authors:** Mariana Fragão-Marques, Rui Vitorino, Isaac Barroso, Inês Falcão-Pires, Adelino Leite-Moreira, Fábio Trindade

**Affiliations:** 1 UnIC@RISE, Department of Surgery and Physiology, Faculty of Medicine of the University of Porto, 4200-319 Porto, Portugal; rvitorino@ua.pt (R.V.); ipires@med.up.pt (I.F.-P.); afleitemoreira@gmail.com (A.L.-M.); ftrindade@med.up.pt (F.T.); 2 Department of Clinical of Clinical Pathology, São João University Hospital Centre, 4200-319 Porto, Portugal; isaacbarroso@gmail.com; 3 iBiMED–Institute of Biomedicine, Department of Medical Sciences, University of Aveiro, 3810-193 Aveiro, Portugal

**Keywords:** atrial fibrillation, aortic stenosis, cardiac surgery, biomarker, proteomics, mass spectrometry, pericardial fluid

## Abstract

Atrial fibrillation (AF) is the most common arrhythmia with adverse clinical outcomes. Pericardial fluid (PF) mirrors the heart’s pathophysiological status due to its proximity. This study aimed to characterise the PF proteome to identify new biomarkers of disease. Eighty-three patients submitted to aortic valve replacement surgery with severe aortic stenosis were selected, and their baseline echocardiographic and clinical variables were documented. Thirteen samples were selected blindly for proteome characterisation following a shotgun (GeLC–MS/MS) and a label-free quantification approach (LFQ). According to previous AF history, a partial least squares discriminant analysis (PLS-DA) was conducted, and the top 15 variables important in projection were identified. To inquire potential biomarkers, ROC curves were designed using LFQ data. Target proteins were further validated by ELISA, in both pericardial fluid and serum. Proteome analysis uncovered nine proteins up- and downregulated ≥2-fold. Annexin A1, annexin A2, and vimentin were among the top 15 most important variables for group discrimination in PLS-DA. Protein—protein interaction and gene ontology enrichment analysis presented functional interaction among identified proteins, which were all part of focal adhesion sites. Annexin A1 was increased in the pericardial fluid of AF patients but not in serum when quantified by ELISA. Annexin A1 is a novel pericardial fluid biomarker of AF in patients with severe aortic stenosis.

## 1. Introductions

Atrial fibrillation (AF) is the most common arrhythmia with clinical significance, with increased risk of thrombosis, heart failure, and mortality, including in adjusted models [1]. Aortic stenosis-induced left ventricle (LV) hypertrophy often results in decreased compliance and higher LV end-diastolic pressure, leading to increased left atrium (LA) afterload and enlargement [2]. LA dilatation shortens the atrial refractory period, favouring the occurrence of ectopic firing that initiates and maintains AF. Myocardial hypertrophy may contribute to AF pathophysiology through abnormal calcium handling, causing ectopic triggers from delayed afterdepolarisations [3]. Recently, LA fibrosis and collagen type III gene expression were found increased in AF patients with AS, although the tissue inhibitor of metalloproteases (TIMPs) 1 and matrix metalloprotease (MMP) 16/TIMP4 ratio gene expression were decreased, suggesting extracellular matrix remodelling in this subset of patients [4]. Proteome analysis through mass spectrometry (MS) improved current medical research by allowing the identification of a large spectrum of proteins at once, excusing the need of mining scientific literature to select hypothetical biomarkers of a given disease. Indeed, proteomics has been used to identify markers of disease in AF patients submitted to cardiac surgery, namely, collagen type I, type III, RAP1, LRG1, and fibulin-1 [5,6]. Additionally, patients with AF and severe rheumatic valve disease presented an altered expression of desmoplakin, COX5b, and HSPβ1 in a previous work [7]. However, to the best of our knowledge, biomarkers of AF in the setting of AS are yet to be identified, which could help stratify patients according to the risk of developing short- and long-term outcomes after surgery or in AS in general. Furthermore, classical biomarkers of AS severity, such as aortic valve maximum and mean gradients, are reduced in AF, with markers associated with fibrosis and remodelling predicting worse outcomes [4]. Moreover, former studies have low sample size, either in proteome discovery phase or in the subsequent validation stage, usually by Western blotting. Although serum and atrial tissue are the most common biological matrices tested in this context, pericardial fluid is easily obtained during cardiac surgery or pericardiocentesis, with the benefit of concentrating heart-derived factors, thus being a promising biological fluid for identifying relevant therapeutic targets and for disease monitoring [8]. Recently, pericardial fluid growth/differentiation factor 15 levels were associated with cardiac and kidney function in patients with coronary artery disease [9], although pericardial fluid remains underrated and poorly studied, particularly in patients with severe AS undergoing aortic valve replacement (AVR). This study, thus, aimed to analyse the pericardial fluid proteome of AF patients with severe aortic stenosis by MS and to identify biomarkers of disease.

## 2. Material and Methods

This study aimed to explore new atrial fibrillation pericardial fluid biomarkers in aortic stenosis, through MS-based proteome identification, and further validation of target proteins, in both pericardial fluid and serum samples.

### 2.1. Study Design

Between 2014 and 2019, 83 patients submitted to both combined (6.7%) and isolated (93.3%) AVR surgery with severe aortic stenosis were selected, and their baseline data concerning echocardiographic parameters and clinical variables were documented. These patients are a part of a larger cohort, so this study represents a post hoc analysis of a prospective cohort. Samples of pericardial fluid and serum were collected and stored (see below). Patients with severe aortic regurgitation were excluded from the study, as were reoperation and emergent cases. Thirteen pericardial fluid samples were randomly selected for proteomic analysis and were further divided according to cardiac rhythm: AF or sinus rhythm (SR; *n* = 8 and *n* = 5, respectively). The surrogate biomarkers identified by MS were tested in a validation cohort, which included 59 recruited patients (AF N = 15 and SR N = 44, respectively). In addition, validated biomarkers were quantified in the serum of 40 patients (AF *n* = 4 vs. SR *n* = 36).

The protocol was approved by the institution’s ethics committee (approval number 35-17), and data confidentiality was ensured. All participants gave written informed consent. The project is in agreement with the Declaration of Helsinki.

### 2.2. Data Collection

Symptoms of heart failure were classified according to the New York Heart Association (NYHA) functional scale, and a diagnosis of coronary artery disease (CAD) was given to patients showing a stenosis >50% on invasive coronary angiography at least on one vessel and/or to patients that have had a previous myocardial infarction. When patients presented a history of stroke or transient ischemic attack and/or presented a stenosis >50% on carotid Doppler ultrasonography, cerebrovascular disease was considered.

Echocardiography was performed by experienced operators up to 6 months prior to surgery, LV ejection fraction was accessed by the modified Simpson rule from biplane 4 and 2 chamber views, and cardiac chamber dimensions were measured as recommended [10]. According to the European Society of Cardiology guidelines, reduced ejection fraction was considered when below 40% [11]. LA enlargement was defined by a left atrium diameter equal or superior to 40 mm.

All patients had an electrocardiogram (EKG) performed up to 6 months before surgery, and AF was defined according to international guidelines as absolutely irregular RR intervals and no discernible, distinct P waves, with an episode lasting at least 30 s being the threshold for diagnostic purposes [12]. 

### 2.3. Pericardial Fluid and Serum Sample Collection

Whole blood samples were collected from a peripheral vein in tubes with a clot activator and separation gel before surgical intervention, and pericardial fluid was collected at the beginning of surgery during pericardial section. Both types of samples were centrifuged at 870 g, 4 °C, for 10 min to remove cellular components, and the supernatants were stored at −80 °C for posterior proteome analysis or enzyme-linked immunosorbent assay (ELISA). Protein concentration was estimated by the DC protein assay (BioRad^®^), using BSA as standard.

### 2.4. Fractionation of Pericardial Fluid Proteins

Thirteen pericardial fluid samples (AF *n* = 8 vs. SR *n* = 5) were assigned for MS analysis. In order to remove excess albumin, the proteome was fractionated with EDTA-functionalised magnetic nanoparticles (NPs@EDTA), as described in [13]. Briefly, 3 mg of protein was incubated with 1 mg of NPs@EDTA in continuous agitation for 1 h at room temperature. Subsequently, the albumin-rich supernatant was removed, and the beads were washed 3 times with 500 μL of MES buffer (0.01 M 2-(N-morpholino)ethanesulfonic acid, 0.01 M NaCl, pH 6.5–8.5) for 5 min. To elute the desired albumin-poor fraction, the beads were incubated with 30 μL of Laemmli loading buffer and agitated for 10 min. Proteins dissolved in loading buffer were separated by SDS-PAGE. Gels were then incubated in fixation solution (methanol: acetic acid 40:10 for 45 min, stained with colloidal Coomassie blue G250, and distained with 20% methanol until an optimal contrast was achieved.

### 2.5. Protein Analysis by NanoHPLC-MS/MS

Each sample lane was cut in 16 pieces and washed with 100 mM NH_4_HCO_3_ and acetonitrile. Proteins were then reduced with 10 mM dithiothreitol (30 min, 60 °C), alkylated in the dark with 55 mM iodoacetamide (30 min, 25 °C), and washed again with 100 mM NH_4_HCO_3_ and acetonitrile, before being digested with trypsin overnight (37 °C). Peptides were extracted first with 10% formic acid (FA) and with FA 10%/acetonitrile (1:1), and afterwards vacuum-dried. Next, peptides were resuspended in 2% acetonitrile and 1% FA and purified using OMIX Tip C18 columns (Agilent), following the manufacturer’s instructions. Peptides were redried and kept frozen (−80 °C) until MS-based analysis.

The peptide mixtures were resuspended in 20 μL loading solvent (0.1% trifluoroacetic acid in water/acetonitrile, 98/2). An amount of 10 μL of the peptide mixtures was analysed by an LC−MS/MS system on an UltiMate 3000 RSLC nano LC (Thermo Fisher Scientific, Bremen, Germany) in-line connected to a Q Exactive HF mass spectrometer (Thermo Fisher Scientific). Peptides were first loaded on a trapping column (made in-house, 100 μm ID × 20 mm, 5 μm beads C18 Reprosil-HD, Dr. Maisch, Ammerbuch-Entringen, Germany), and after flushing from the trapping column, peptides were loaded on an analytical column (made in-house, 75 μm ID × 400 mm, 1.9 μm beads C18 Reprosil-HD, Dr. Maisch) using a nonlinear 150 min gradient of 2–56% solvent B (0.1% FA in water/acetonitrile, 20/80 (*v*/*v*)) at a flow rate of 250 nL/min. This step was followed by a 10 min wash reaching 99% solvent B and re-equilibration with solvent A (0.1% FA in water). Column temperature was kept constant at 50 °C (CoControl 3.3.05, Sonation).

The mass spectrometer was operated in data-dependent acquisition, positive ionisation mode, automatically switching between MS and MS/MS acquisition for the 16 most abundant peaks in a given MS spectrum. The source voltage was set to 2.8 kV, and the capillary temperature was 250 °C. One MS1 scan (*m*/*z* 375–1500, auto gain control target 3E6 ions, maximum ion injection time of 60 ms) acquired at a resolution of 60,000 (at 200 *m*/*z*) was followed by up to 16 tandem MS scans (resolution of 15,000 at 200 *m*/*z*) of the most intense ions fulfilling predefined selection criteria (automatic gain control target of 1E5 ions, maximum ion injection time of 80 ms, isolation window of 1.5 *m*/*z*, fixed first mass of 145 *m*/*z*, spectrum data type: centroid, under fill ratio 1%, intensity threshold of 1.3E4, exclusion of unassigned singly charged precursors, peptide match preferred, exclude isotopes on dynamic exclusion time of 12 s). The higher collision energy dissociation was set to 28% normalised collision energy, and the polydimethylcyclosiloxane background ion at 445.12002 Da was used for internal calibration (lock mass).

### 2.6. Protein Identification

Data analysis was performed with MaxQuant (version 1.6.1.0) using the Andromeda search engine (Max Plank Institute of Biochemistry, Martinsried, Germany). Spectra were searched against the human proteins in the Swissprot database (release March 2018). The mass tolerance for precursor and fragment ions was set to 20 and 4.5 ppm, respectively, during the main search. Enzyme specificity was set to C-terminal to arginine and lysine, also allowing cleavage at arginine/lysine—proline bonds with a maximum of two missed cleavages. Carbamidomethylation on cysteines was set as a fix modification. Variable modifications were set to oxidation of methionine, acetylation of protein N-termini, and phosphorylation of serine, threonine, or tyrosine. Matching between runs was allowed using a 0.7 min match time window and a 20 min alignment time window. Proteins were quantified by the MaxLFQ algorithm integrated in the MaxQuant software. Identified peptides were filtered using a 1% false discovery rate. Only proteins identified with 2 or more peptides were considered. 

### 2.7. Bioinformatics Analysis

Dysregulated proteins were assessed by calculating the fold difference between proteins quantified in the pericardial fluid of SR and AF patients. An unpaired two-tailed *t*-test was used to filter significant differences.

Significantly dysregulated proteins were the object of protein—protein interaction and functional enrichment analyses with a STRING (version 10.5) webtool. A score of 0.4 was set as the minimum threshold for consideration of the validated and putative interactions.

A partial least squares discriminant analysis was performed with MetaboAnalyst (version 4.0, McGill campus, Montreal, QC, Canada) as a final inquiry of the potential of a given protein biomarker. To do that, protein MaxQuant intensities were uploaded, log2-transformed, and autoscaled. Protein importance concerning the distinction between AF and SR patients was then evaluated through the analysis of variable importance to projection (VIP score). 

### 2.8. Pericardial Fluid and Serum Protein Quantification

A total of 59 patients (AF *n* = 15 vs. SR *n* = 44) were selected for assessing pericardial fluid proteins identified by MS. ELISA assays were performed following the manufacturer’s instructions (Elabscience, E-EL-H5512 Human ANXA1 (annexin A1) ELISA Kit, E-EL-H10448 Human ANXA2 (Annexin A2) ELISA Kit, and E-EL-H1094 Human VIM (Vimentin) ELISA Kit). 

Annexin A1, as a potential biomarker candidate confirmed by ELISA, was further quantified in the serum of 40 patients (AF *n* = 4 vs. SR *n* = 36) through the same quantitative method (Elabscience, E-EL-H5512 Human ANXA1). Serum samples were from the same prospective cohort of patients, with approximately 63% of participants having both pericardial fluid and serum samples.

### 2.9. Statistical Analysis

Continuous variables were represented as mean and standard deviation (sd), according to normality testing, and categorical variables as percentages. A *t*-test for independent samples was used to compare means of continuous variables, and an χ-squared or Fisher’s exact tests were used for the comparison of categorical variables. Linear regression was performed to test correlations between continuous variables. Results are presented as β coefficients, 95% confidence intervals (CI), and *p* values. Statistical significance was considered when *p* < 0.05. IBM SPSS Statistics version 25 was used for all statistical analyses.

## 3. Results

### 3.1. Demographics of Patients from the Discovery Phase (Proteomics)

The pool of patients analysed by MS was similar regarding their baseline characteristics (Table 1). AF patients had a mean age of 73.50 ± 7.78 (mean ± sd), while SR patients were younger, with a mean of 64.00 ± 16.05 years (*p* = 0.175). Males represented 50.0% of the AF patients and 40.0% of the SR participants. Comorbidities were balanced between both groups, with hypertension being the most representative in either group (87.5% in AF vs. 60.0% in SR, *p* = 0.510). Symptoms of heart failure were present in 75.0% vs. 50.0% (*p* = 0.547) of the participants, respectively. LA dilation was extremely common, representing 85.7% in the AF subset and 100% in the SR subgroup (*p* = 0.462). The AF and SR patients were not significantly different with regard to AS severity, according to maximal (*p* = 0.743) and mean transvalvular pressure gradient (*p* = 0.788), as well as valve area (*p* = 0.950) criteria.

### 3.2. Identification of Atrial Fibrillation Surrogate Markers through Pericardial Fluid Proteomics Analysis

**Figure 1:**
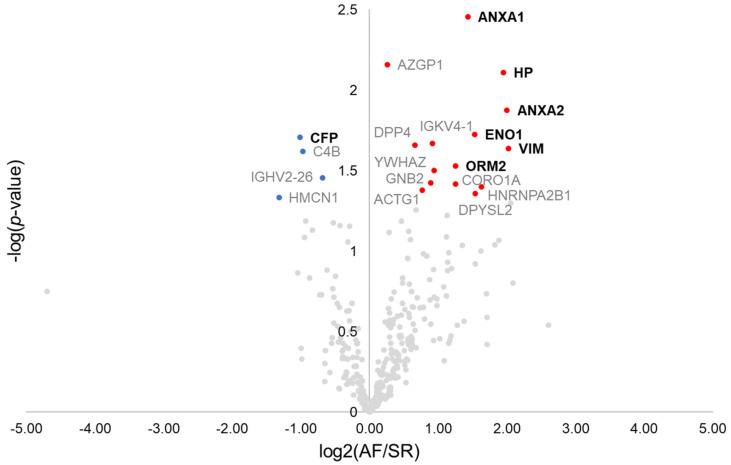
Volcano plot showing dysregulated proteins in the pericardial fluid of patients with atrial fibrillation. Red and blue dots mark, respectively, proteins significantly increased and decreased in patients with atrial fibrillation. Proteins quantified in all patients and with ≥2-fold change are marked in bold.

Overall, 770 proteins were identified in the pericardial fluid of the 13 patients composing the discovery cohort (Supplementary Materials Table S1). As a quality control step, the correlation of each patient’s pericardial fluid proteome to the remaining patient’s proteome was analysed, which identified one control individual as a potential source of bias (Supplementary Materials Figure S1). Therefore, such patient was excluded, with the final discovery population comprising 8 AF patients and 4 SR controls. The first step towards the identification of surrogate markers for AF was to browse dysregulated proteins by the means of a *t*-test (Figure 1). Nineteen dysregulated proteins were found, from which 11 displayed a ≥2-fold difference between groups. These included the protein properdin (CFP, −2.0-fold), significantly downregulated in AF, and the proteins annexin A1 (ANXA1, +2.7-fold), annexin A2 (ANXA2, +4.0-fold), haptoglobin (HP, +3.9-fold), alpha-enolase (ENO1, +2.9-fold), vimentin (VIM, +4.1-fold), and alpha-1-acid glycoprotein 2 (ORM2, +2.4-fold), all significantly upregulated in AF. These 7 proteins could be successfully quantified in all samples. The remaining proteins are identified in the volcano plot through the respective gene name (Figure 1).

**Figure 2:**
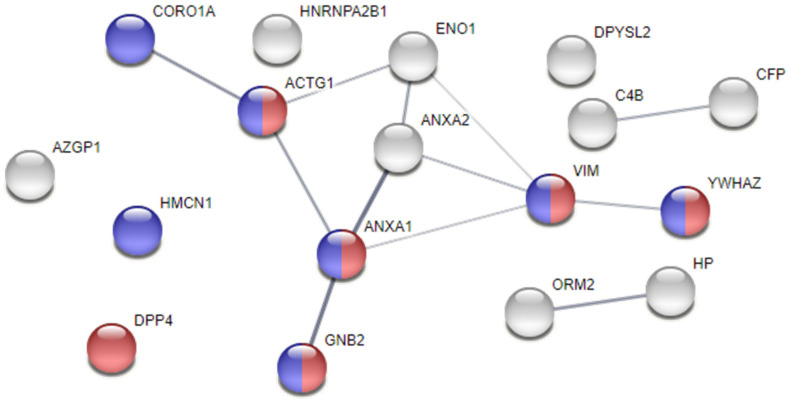
Protein–protein interaction analysis of dysregulated proteins in the pericardial fluid of atrial fibrillation subjects. Analysis performed with STRING. The thickness of the edges represents the confidence of the interaction. Proteins are identified with the respective gene name. Blue nodes identify proteins localised in cell junctions, while red nodes identify proteins present in focal adhesions.

Moreover, a protein—protein interaction and functional enrichment analyses were conducted in a STRING webtool, aiming at exploring the relationships between proteins and those that, by looking at the deemed cellular localisation or to the attributed biological processes, would be significant players in AF pathogenesis. As shown in Figure 2, the proteins ANXA1, ANXA2, ENO1, and VIM, in addition to γ-actin (ACTG1), coronin-1A (CORO1A), guanine nucleotide-binding protein G(I)/G(S)/G(T) subunit β-2 (GNB2), and 14-3-3 protein ζ/δ (YWHAZ), cluster up, showing higher probability of interaction. Noticeably, we found 7 proteins localised in cell junctions and 6 specifically localised in focal adhesions, whose integrity is key to maintain normal electrical activity propagation in syncytium. The increased levels of ANXA1, ANXA2, and VIM in the pericardial fluid of AF patients might mark loss of atrial tissue integrity, triggering arrhythmic events.

**Figure 3:**
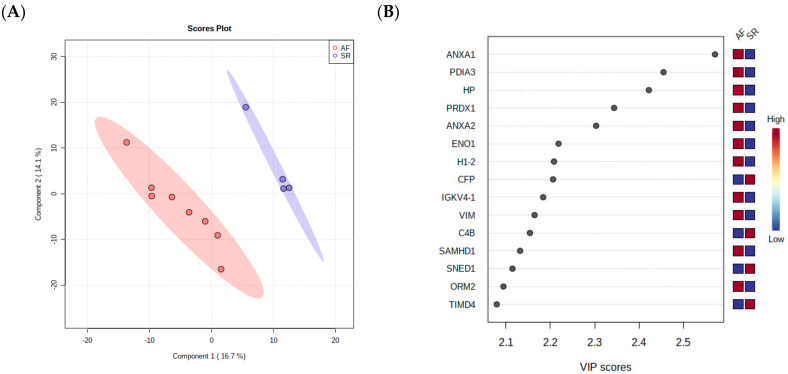
(**A**) PLS-DA analysis, showing that pericardial fluid proteome holds value in the identification of ill patients. (**B**) The most important proteins for the separation of patients in the first component are arranged from top to bottom according to the importance to data projection (variable importance in projection, VIP score).

As a final step towards the selection of surrogate markers for validation, a PLS-DA was performed, as this analysis takes into consideration not only the relative changes in protein levels between the groups but also the correlation of different proteins, ultimately reducing data dimensionality to a few components. The separation of AF and SR in 2 components is clear in Figure 3A, showing that pericardial fluid proteome holds value in the identification of ill patients. The most important proteins for the separation of patients in the first component are depicted in Figure 3B, where they are arranged from top to bottom according to the importance to data projection (variable importance in projection, VIP score). ANXA1 pops up as the most important variable to discriminate patients. ANXA2 and VIM, two highly interconnected proteins according to STRING analysis, are also among the top 10 most important variables to discriminate the two conditions. Of note, the protein disulfide-isomerase A3 (PDIA3), an important player in the response to endoplasmic reticulum stress and involved in the activation of the extrinsic apoptotic pathway; the acute-phase response reactant haptoglobin (HP); and peroxiredoxin-1 (PRDX1), an essential protein for fighting oxidative stress, were also deemed important to differentiate AF from SR.

Based on these three analyses and on quality criteria for protein identification (i.e., number of unique peptides identified and sequence coverage), we selected ANXA1 (14 peptides, 47.1% coverage), ANXA2 (21 peptides, 63.7% coverage), and VIM (35 unique peptides, 66.1% coverage) for the validation of their biomarker value in an independent cohort. 

### 3.3. Demographics of Patients from the Validation Cohort

#### Pericardial Fluid

The validation cohort was balanced concerning baseline characteristics (Table 2). AF patients were older when compared with their SR counterparts, although not statistically significant—73.33 ± 9.63 vs. 69.02 ± 9.38, *p* = 0.132. Males were 46.7% of the AF subgroup, but 52.3% of the SR participants (*p* = 0.708). Hypertension remained the most common comorbidity (AF 11 (73.3%) vs. SR 35 (83.3%), *p* = 0.400), followed by diabetes mellitus (5 (33.3%) vs. 12 (28.6%), *p* = 0.729). Heart failure NYHA class superior or equal to II represented 71.4% vs. 76.9% of the patients, respectively (*p* = 0.682). Aortic valve gradients were lower in the AF patients, although nonsignificant (maximum gradient *p* = 0.287, mean gradient *p* = 0.296). Additionally, serum samples were balanced between groups regarding patient characteristics (Table 3), with a mean age of 76.75 ± 7.63 vs. 71.47 ± 8.34 in the AF and SR patients, respectively (*p* = 0.235). The most common comorbidity remained to be hypertension—AF 3 (75.0%) vs. SR 26 (72.2%), *p* = 1.000.

### 3.4. Pericardial Fluid Quantification of Annexin A1, Annexin A2, and Vimentin

**Figure 4:**
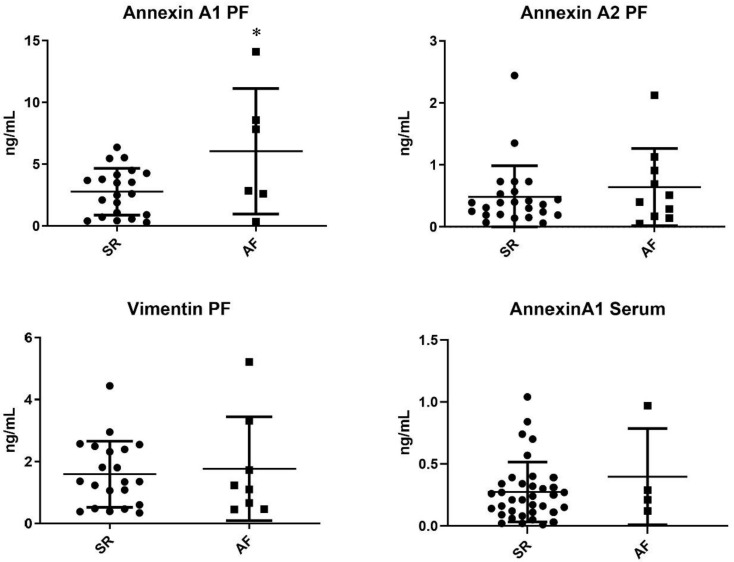
ELISA pericardial fluid quantification of proteins dysregulated in the arrhythmic group (AF vs. SR) * *p* < 0.005.

Target proteins resulting from proteome analysis were quantified in the pericardial fluid of patients from the validation cohort. Annexin A1 was significantly increased in AF patients when compared with SR patients (6.05 ± 2.07 ng/mL vs. 2.78 ± 0.41 ng/mL, *p* = 0.020). Moreover, annexin A2 was, on average, increased in the AF subgroup, although without reaching significance—0.64 ± 0.20 ng/mL vs. 0.48 ± 0.10 ng/mL, *p* = 0.442. However, no differences in vimentin expression were observed—1.77 ± 0.59 vs. 1.59 ± 0.23, *p* = 0.736 (Figure 4).

### 3.5. Serum Quantification of Annexin A1

Annexin A1 was further quantified in the serum of patients from the respective validation cohort. The target protein did not present significantly different concentrations between groups—AF 0.40 ± 0.39 ng/mL vs. 0.27 ± 0.24 ng/mL (Figure 4).

### 3.6. Annexin A1 and Aortic Stenosis Severity

Annexin A1 was not associated with the presence of heart failure symptoms (NYHA class ≥ II)—β 0.07 (−0.051–0.065), *p* = 0.812. Moreover, neither the aortic valve maximum gradient nor the mean gradient changed according to annexin A1 quantifications—β 0.204 (−2.24–2.65), *p* = 0.863; β −0.036 (−1.47–1.40), *p* = 0.959. The aortic valve area remained similar regardless of the pericardial fluid protein concentration—β −0.17 (−0.41–0.007), *p* = 0.147.

## 4. Discussion

The analysis of the pericardial fluid proteome through a state-of-the-art MS instrument identified annexin A1, annexin A2, and vimentin as potential biomarkers of AF in severe aortic stenosis. Gene ontology enrichment analysis revealed these proteins to be part of focal adhesions, important structures for the heart’s electromechanical coupling. These proteins were among the top 10 most important proteins to separate AF from SR patients in a multivariate analysis. Therefore, we selected these for validation. In a larger validation cohort, annexin A1 showed a significant increase in the pericardial fluid of AF patients, although no significant differences were found in serum.

Pericardial fluid vimentin was not increased in AF in this cohort, confirming results by Lungenbiel et al., in which pigs with induced AF did not present differences in vimentin-expressing inactive fibroblasts [14]. Likewise, annexin A2 remained similar between AF and SR patient subgroups. This protein has been identified as a marker of increased coronary artery calcification and epicardial adipose tissue volume [15], although a further study did not find any differences in annexin A2 between aortic stenosis and insufficiency patients, after confirming mass spectrometry results by Western blotting [16].

Annexin A1 levels were found significantly higher in AF patients’ pericardial fluid. This protein is present in inflammatory cells, such as macrophages and lymphocytes, and it is produced when there is an inflammatory stimulus. It can inhibit PLA2, as well as macrophage-induced NO synthase [17,18]. Furthermore, annexin A1 decreases TNF-α and IL-6, inhibiting macrophage and monocyte migration [17]. In a normal setting, most annexin A1 is located in the cell cytoplasm, while with inflammation the protein moves to the cell surface, interacting with adhesion molecules, promoting neutrophil shedding from endothelial cells. Annexin A1 can activate ERK1/2 and competes with VCAM-1 for binding to integrin a4b1, thus inhibiting the adhesion of inflammatory cells to the endothelial wall [19]. It is induced by glucocorticoids and regulates the activity of T cells by interfering with TCR signalling pathways and ERK–MAPK [20]. Moreover, this protein can further reduce inflammation by promoting phagocytosis of apoptotic inflammatory cells [20]. Increased levels of annexin A1 have been reported in acute idiopathic pulmonary fibrosis and liver fibrosis [21], while in COPD it promotes lung fibroblast proliferation, migration, and differentiation through ERK1/2 and p38 signalling [22]. Annexin A1 has been associated with worse congestion, higher creatinine elevation, and increased morbidity and mortality in acute heart failure [23]. This protein is also a substrate for PDGF, which can activate atrial fibroblasts, which, in turn produce fibrogenic molecules, causing fibrosis. Activation of a fibroblast TRPC3 channel promotes calcium entry to atrial fibroblasts, with ERK activation and phosphorylation, which enhances fibroblast survival and fibrosis [24]. This structural remodelling, in turn, is central to most AF subtypes, altering cardiomyocyte electrical coupling due to misplacing and changing the structure of connexins, promoting fragmented electrical conduction [25]. Indeed, an increased afterload, such as that seen in severe aortic stenosis, has been associated with myocardial fibrosis and AF [26]. Hence, annexin A1 has a ubiquitous anti-inflammatory action, targeting several aspects of the immunologic response mechanism, although it appears to promote fibrosis. Annexin A1 could contribute to AF pathophysiology in aortic stenosis and function as a countermeasure of inflammation, thus possibly representing a marker of disease progression. 

There is a lack of biomarkers in AF, for either disease progression or disease-related outcomes. This study is the first to identify annexin A1 as a marker of AF in aortic stenosis and in the pericardial fluid. However, there were no differences in annexin A1 concentration between groups in serum samples, which could be explained by either a low number of patients or a significant difference between pericardial fluid and serum concentrations of this protein. Further research is necessary to confirm these results and further test this biomarker as a marker of AF progression and outcomes, such as stroke and all-cause mortality. Additionally, plenty of studies concerning proteome analysis by MS have a very low number of patients, without a validation cohort with a higher number of subjects. Our study, although with a relatively low number of samples for spectrometric analysis, has a significant number of patients in which the validation was conducted. Several research papers validate the protein targets through Western blotting; in this work, we opted for an ELISA, as it is more directly applicable in clinical practice, easily implemented and automated in a clinical laboratory setting. This study also demonstrates the usefulness of pericardial fluid as a biological matrix for the identification of biomarkers for cardiac diseases.

## 5. Conclusions

Annexin A1 is a novel pericardial fluid biomarker of AF in patients with severe aortic stenosis.

## Figures and Tables

**Table 1:** Baseline characteristics of patients analysed by mass spectrometry.

Variable (*n*(%))	SR (*n* = 5)	AF (*n* = 8)	*p* Value
Age, years (mean ± sd)	64.00 ± 16.05	73.50 ± 7.78	0.175
Gender (Male)	2 (40.0)	4 (50.0)	1.000
Smoking	0 (0)	0 (0)	-
Diabetes Mellitus (Type 2)	1 (20.0)	3 (37.5)	1.000
Hypertension	3 (60.0)	7 (87.5)	0.510
Previous AMI	0 (0)	0 (0)	-
Coronary Artery Disease	0 (0)	1 (12.5)	1.000
Cerebrovascular Disease	0 (0)	0 (0)	-
NYHA Class ≥ II	2 (50.0)	6 (75.0)	0.547
Angina	0 (0)	1 (14.3)	1.000
Syncope/Lipothymia	0 (0)	0 (0)	-
Ejection Fraction (<40%)	0 (0)	1 (14.3)	1.000
Left Atrium Dilation (LAD > 40 mm)	4 (100)	6 (85.7)	1.000
AoV MaxG, mmHg (mean ± sd)	83.40 ± 20.29	88.43 ± 28.34	0.743
AoV MeanG, mmHg (mean ± sd)	50.40 ± 10.55	52.86 ± 17.53	0.788
AoV Area, cm^2^ (mean ± sd)	0.73 ± 0.21	0.73 ± 0.20	0.950

**Table 2:** Patient characteristics of the validation population for identified proteome targets (pericardial fluid).

Variable (*n*(%))	SR (*n*= 44)	AF (*n* = 15)	*p*-Value
Age, years (mean ± sd)	69.02 ± 9.38	73.33 ± 9.63	0.132
Gender (male)	23 (52.3)	7 (46.7)	0.708
Smoking	6 (14.3)	0 (0)	0.325
Diabetes mellitus (type 2)	12 (28.6)	5 (33.3)	0.729
Hypertension	35 (83.3)	11 (73.3)	0.400
Previous AMI	2 (4.8)	0 (0)	1.000
Coronary artery disease	8 (19.0)	1 (6.7)	0.420
Cerebrovascular disease	3 (7.1)	1 (7.1)	1.000
NYHA class ≥ II	30 (76.9)	10 (71.4)	0.682
Angina	6 (16.2)	3 (23.1)	0.679
Syncope/lipothymia	4 (10.8)	0 (0)	0.561
Ejection fraction (<40%)	2 (5.4)	1 (8.3)	1.000
Left atrium dilation (LAD > 40 mm)	28 (84.8)	11 (91.7)	0.552
AoV maxG, mmHg (mean ± sd)	85.19 ± 21.45	77.54 ± 21.92	0.287
AoV meanG, mmHg (mean ± sd)	52.82 ± 12.77	48.31 ± 13.97	0.296
AoV area, cm^2^ (mean ± sd)	0.75 ± 0.16	0.76 ± 0.15	0.884

**Table 3:** Patient characteristics of the validation population for annexin A1 (serum).

Variable (*n*(%))	SR (*n* = 36)	AF (*n* = 4)	*p*-Value
Age, years (mean ± sd)	71.47 ± 8.34	76.75 ± 7.63	0.235
Gender (male)	23 (63.9)	1 (25.0)	0.283
Smoking	3 (8.6)	0 (0.0)	1.000
Diabetes mellitus (type 2)	9 (25.0)	0 (0.0)	0.557
Hypertension	26 (72.2)	3 (75.0)	1.000
Coronary artery disease	3 (9.1)	0 (0.0)	1.000
Cerebrovascular disease	1 (3.1)	0 (0.0)	1.000
NYHA class ≥ II	22 (78.6)	3 (100.0)	1.000
Angina	4 (14.8)	0 (0.0)	1.000
Syncope/lipothymia	3 (11.1)	0 (0.0)	1.000
Left atrium dilation (LAD > 40 mm)	16 (66.7)	3 (100.0)	0.532
AoV maxG, mmHg (mean ± sd)	80.30 ± 22.54	83.00 ± 2.94	0.815
AoV meanG, mmHg (mean ± sd)	50.79 ± 14.01	53.50 ± 6.86	0.709
AoV area, cm^2^ (mean ± sd)	0.77 ± 0.18	0.65 ± 0.21	0.346

## Data Availability

The proteome data have been deposited to the ProteomeXchange Consortium via the PRIDE partner repository, with the dataset identifier PXD015607.

## Supplementary Materials

The following supporting information can be downloaded at: https://www.mdpi.com/article/10.3390/jpm12020264/s1.
