# Supplementary material for: Pericardial Fluid Annexin A1 Is a Marker of Atrial Fibrillation in Aortic Stenosis: A Proteomics Analysis
Source: J Pers Med. 2022 Feb 11;12(2):264. doi: 10.3390/jpm12020264 (PMC8880366; doi:10.3390/jpm12020264)
Supplement: Supplementary file 1 [file jpm-12-00264-s001.zip › jpm-1564630- supplementary.pdf]

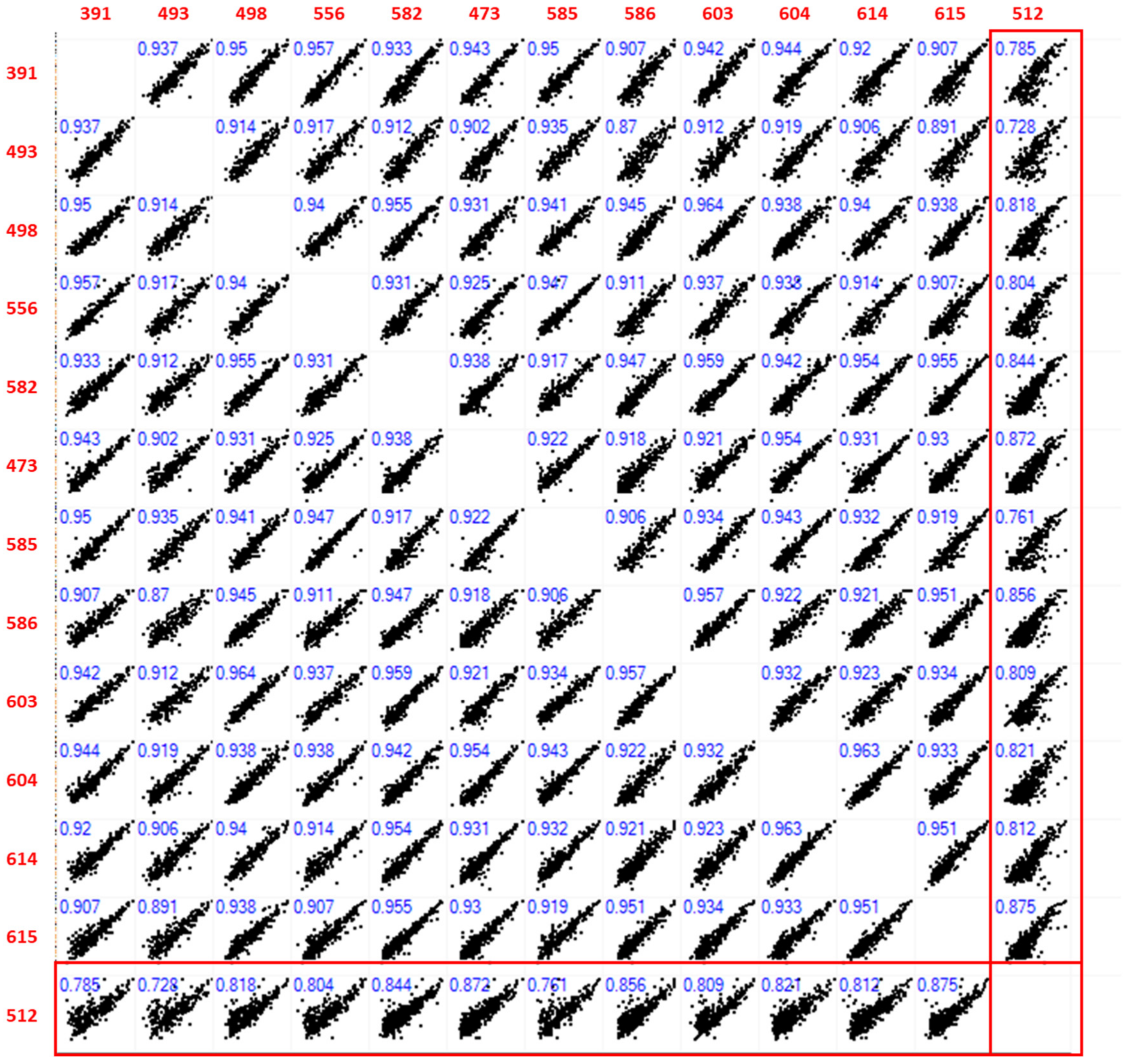

Supplementary Figure S1. Pericardial fluid proteome correlation between all 14 samples. Pearson's correlation coefficient is given for each pair. The sample #512 stands out as being a potential outlier with correlation coefficients spanning from 0.728-0.875. In any other pair of samples the minimal correlation coefficient is 0.87. with all but two cases, above 0.90.

Supplementary Table S1.

PROTEIN ID		1	2	3	4	5	6	7
P04083	ANXA1	25.67659	27.22757	27.52462	26.25321	26.35792	26.93607	26.47923
P25311	AZGP1		23.36333	23.38007				23.40587
P00738	HP	30.33117	31.02175	30.22516	29.7037	31.30986	29.99159	28.35191
P07355	ANXA2	24.858	27.17198	26.18331	27.2606	27.01542	28.26446	25.45168
P06733	ENO1	26.42446	28.39411	26.89111	28.27427	28.75306	27.24173	27.53148
P27918	CFP	28.24762	26.90564	27.7888	28.74814	27.30095	28.02538	28.3748
P06312	IGKV4-1	28.4466	27.89383	28.14438	27.37401	27.24127	27.85739	27.8725
P27487	DPP4	24.59012	24.68998	24.13579	25.35175	24.4617	24.32492	
P08670	VIM	29.03486	29.25343	28.46135	29.90081	29.91428	31.98053	28.86124
P0C0L5	C4B	29.33264	28.16029	27.92897	29.72359	28.96873	28.96441	29.09388
P19652	ORM2	30.04726	28.80904	28.57127	28.19178	28.83681	27.91336	27.17969
P63104	YWHAZ	24.75119	24.87479	24.38304	24.26942	25.64612	24.75644	
A0A0B4J1	IGHV2-26		24.98715		24.34489	24.37292		
P62879	GNB2		23.91296		24.08497	24.28842	23.90867	
P31146	CORO1A		26.32833	25.72005	25.76154	26.46524	27.36311	25.60549
P22626	HNRNPA2B1		26.2781	25.44474	25.74819	27.56245	26.47449	26.50355
P63261	ACTG1	30.76833	31.5965	30.70553	31.02347	31.9303	30.87621	31.01996
Q16555	DPYSL2		26.0633	24.97014	24.52558	27.26472	25.45748	25.46831
Q96RW7	HMCN1			22.05619	22.56533		20.85992	
O43175		26.75124	27.6601	27.95737	27.31916	29.53169		28.19859
P02763		30.58459	30.11682	30.20618	30.50047	31.39647	30.26674	29.63095
P08519		27.81195	28.02136	27.00635	28.97686	27.07572	27.23087	28.35489
P18065		26.5409	27.91519	27.6675	26.99529	27.40682	27.3892	26.95593
P35443		25.36673	26.36928	27.82438	26.61569	26.32437	26.8147	26.14859
P07358		31.97021	32.57808	32.25696	31.94269	32.717	32.83244	32.97141
Q15582		28.77571	29.54309	29.52055	29.79399	29.53647	29.40109	29.90971
O14786		27.5974	28.21623	28.32247	27.90374	28.22177	28.09974	28.23215
P04196		34.93108	32.66774	33.62375	34.22195	33.17947	33.60565	33.89211
A0A075B6K4		26.15064			26.676	26.1564		27.21123
Q9UBX7			25.1752	24.98841		24.83759	25.26546	25.27732
P01876		32.66717	34.9109	33.10548	31.104	31.95099	32.09046	32.5413
P26572					24.25415	24.35447	24.6457	
P02760;CON	P0097	30.2294	29.74644	29.22117	28.92394	29.33322	28.85007	28.32479
P21333;O75369			27.47034	25.95549	27.1756	28.20275	28.2141	26.18775
Q9UJJ9			23.91916		23.49045	23.9664	23.88418	23.90104
Q06033;CON	Q0V8	29.6433		25.72297	25.12416	25.75034	26.09861	25.89864
P28482			25.6666	24.57103	24.39225	26.94179	24.58354	23.90967
Q5VTE0;P68104;Q05		27.13124	29.81975	28.49888	29.23991	30.69154	28.95543	29.08737
P04792		24.50761	27.19732	25.8452	25.6602	27.14385	25.32134	25.8688
P01700;P01699		28.10029	29.02892	29.10372	27.93568	28.62006	27.30348	29.08015
Q16851			26.23188	25.54498	25.2003	26.48948	24.91456	24.69445
P04040		23.74684	24.51551	24.84153	24.61885	23.62845	23.94583	24.84575
P01011		32.96657	31.13596	31.85002	31.11954	31.58781	32.19657	31.87635
P08758			24.26428		24.84168	23.83016	24.9616	
P17655			26.46832	25.56973	25.19535	28.20513	25.90385	25.14511
Q8WUT4		24.70271	25.99591	25.24782	27.96361	26.01965	24.92325	25.87366
A0A087WSY6		26.74855	26.58205	27.41522	26.62296	26.46888	28.81112	28.24717

P08123	24.51953	24.45463	24.60286	24.96724	23.55215	24.06686	24.25479
P14625;Q58FF3	26.94011	24.61717	25.86424	25.41461	25.52304	26.78432	25.36045
P00488	24.06669	24.92909	26.8776	25.08505	24.67788	26.94525	27.04291
P04432;P01597	25.88437	26.32807	26.33258	25.12372	25.9785	26.48281	25.78534
P01602	28.81931	28.63079	29.5689	28.2833	28.57913	28.57315	29.76077
P36222	25.56355	26.80341	27.3232	25.98659	26.65581	26.25951	25.15279
P14618;P30613	27.40154	29.78706	28.6053	28.76882	30.17176	29.23473	29.31872
P30101	24.08991	23.49045	24.70541	25.6871	26.10829	27.87859	24.92407
Q96KN2	24.32038			24.05499			
A0A0B4J1V0	30.30291	30.8273	30.57389	29.62062	30.2157	30.47837	31.86765
P50238		28.35767	26.72988	25.74016	27.02517	25.86247	26.83569
P06681	28.32582	27.86118	28.46898	29.19535	27.83201	28.65554	28.96843
P13796	24.06439	23.63079	25.53148	23.23332	23.98624	23.50142	23.81939
P02042;P02100			24.1467				
Q07954				25.32286	25.11215	25.5625	
Q00610;P53675		22.96506	23.63832	26.38974	24.35897	26.68485	
P48061	23.2946	23.89541	24.2558		23.13041	23.80892	
P07225;CON P0722	29.3004	28.3672	27.87807	29.75726	27.84816	29.09419	29.15289
Q9Y490;Q9Y4G6	24.80528	27.74516	25.27508	26.93618	28.06435	27.81678	25.67487
P68363;P68366;Q9H8	26.88297	28.8871	28.27994	27.66865	29.63488	27.84984	28.49377
P07357	29.66821	31.3078	30.82669	29.99981	30.87394	30.97971	31.64594
P01599	28.30422	28.0774	28.46135	26.73531	27.06116	29.67961	27.35464
P13639	24.46139	27.03905	25.80863	26.85162	28.03821	26.59463	26.1579
P05090	28.18016	28.61831	28.59793	27.81824	28.25539	28.40381	29.06245
P01591	27.81274	29.02144	28.12524	25.79615	26.66574	26.89215	27.93989
P60842		22.88803	23.59218		23.93948	23.23691	
P07360	29.5874	30.76983	30.91178	30.32195	30.95982	31.26986	31.22613
P0C0L4	34.76875	35.24183	35.12928	35.0623	35.41617	34.98473	35.52755
Q14847		26.79377	24.00676	24.44253	25.06217	25.38538	24.60484
P07900;Q14568;Q58F	26.63118	27.06958	26.75761	26.02557	28.33552	26.45671	26.9546
P12259;CON Q2810	26.04922	23.93436	26.72884	25.31098	24.80262	24.40204	
P15169;CON Q2KJ8	27.8597	25.02821	25.40853	25.5171	26.0087	25.65354	24.89911
P02751	34.82771	34.90352	34.44017	35.82015	35.16166	34.30409	33.91385
B9A064;P0CG04	31.17587	31.17468	31.97729	29.68502	31.40177	31.24156	31.00169
P02765;CON P1276	31.68043	30.51935	30.96781	31.01412	30.90196	31.57655	30.88715
P11142;P54652		24.34942	24.6093	24.28588	24.77756	25.19306	24.49363
Q96IY4;CON Q2KIG	30.33958	30.33692	29.96155	30.18388	30.23717	30.3382	30.47421
Q99969	27.9597	28.08937	28.12874	27.50809	28.06599	28.39108	28.19657
P00746	32.61151	32.58819	32.63696	32.06907	32.66187	32.42049	33.16526
P80748	27.41764	26.52389	27.60399	26.49749	27.43237	28.92011	27.05134
P08238;Q58FF7	24.33756	25.1648	24.31948	24.90885	26.61989	24.97762	25.06336
P01033	27.87988	29.32109	29.19239	28.4927	29.40408	29.0872	28.59335
P35555;P35556			25.81169		23.60919	23.95828	24.14445
A2NJV5;A0A075B6S2;A0A0A0M	28.63405				24.12124	24.70483	27.62526
Q9BR76		25.14783	23.25178	22.97691	24.45149	25.67425	
Q14624;CON Q3T05	34.14589	32.30541	33.08082	33.38882	33.05296	33.1833	32.77825
P0DP08;P0DP06;A0A	27.27355	27.93421	27.58032	26.80378	27.19357	28.1899	27.20443
Q9NZP8	24.84513		25.17154	24.40594	24.74924	24.51713	
P35579	23.45475	27.21882	25.91932	28.73441	27.83213	27.53482	25.20743
Q6PL18;Q9ULI0		26.06848				25.81042	26.0122

P03950	25.73903	27.02285	27.08831	26.93258	26.03364	26.48299	26.25552
P52943		26.10328		24.63533	26.0941		25.26023
Q12805	27.71771	28.60922	28.5587	27.40933	28.3525	27.74169	28.72218
P11021	24.41835	23.82639	24.45588	24.33552	25.35246	26.49785	24.8387
P02748;CON Q3MH	33.24983	32.34723	32.66399	32.05824	32.75017	32.91239	33.15875
A0A0C4DH31		31.41584					27.87578
P46940;Q86VI3		26.98479	25.92619	26.45237	27.77712	27.81586	24.98872
P01780;A0A0B4J1V1;	27.36018	29.05367	29.31311	29.16161	29.26316	28.82019	28.00886
P01860	33.56853	34.28912	33.51418	32.7095	33.67072	33.27156	33.78185
P22692	27.23041	26.97989	26.33381	25.97952	25.9564	26.27535	25.8355
P12111	28.31825	28.52511	28.85466	28.52802	28.11927	28.62763	28.39734
P02671;CON P0267	36.05124	34.84034	33.72405	35.09349	34.55642	35.03133	32.24533
O00391	31.35242	31.70837	32.02089	31.71158	31.53696	31.79386	32.54612
P36980	28.41615	28.2415	28.32053	28.50264	27.52013	27.44166	29.57591
P01008	30.24989	31.35237	32.17899	30.89735	31.65139	30.21882	31.1505
P05783;CON H-INV:	24.2637	23.94002	24.02968	26.78943	28.05367	23.15986	24.94145
P07437;Q9BVA1;Q3Z	25.59226	28.02649	26.92298	26.38309	28.08998	26.27906	27.13575
P08185	26.68953	24.78531	24.75313	25.64058	24.16461	26.27515	24.98076
P22105;Q16473	24.43173		24.7759	24.39277		23.80725	
Q6UWP8	24.50009	25.09599		23.85899	25.2183	24.48481	24.12747
P02747	31.56991	30.1919	29.45421	31.01797	28.81192	30.60505	30.46937
P00734;CON P0073	31.72094	30.46071	30.98617	29.62116	30.50209	30.07176	31.13651
Q9UEW3	26.36274	25.29292		26.87725	25.16258	26.33533	26.42945
P11940;Q9H361;Q4VXU2		25.38024	24.52175	23.88026	26.50127	24.46133	24.34489
P55268				24.46101	23.10385	23.75011	
P14923;P35222		26.32457		23.10843	24.12605	25.63181	
P07858						26.36523	24.42662
P00748	33.57441	32.9118	33.17761	33.28306	33.18013	33.33698	33.74023
Q00839		24.69795	24.74781	25.15588	26.99291	25.38446	24.99347
P81605	26.1043	25.98303	27.07592	25.93337	27.87256	26.56999	
P62826	24.24612	25.98887		24.96878	26.30091	25.16135	25.53481
P35858	31.69449	31.18907	31.22625	29.48633	31.93758	30.79413	31.64629
P20062		23.91095	23.76091				
P01854	23.73128	26.31959	24.47599				
P23142;CON	29.53053	30.04375	31.22147	30.1973	30.55874	30.15714	29.92988
Q9Y279	23.53315	23.91588			23.96323	24.6621	23.55145
P03951	29.88851	29.4941	29.94126	29.4071	29.82514	29.26653	29.99429
P23528;Q9Y281	24.60139	25.03397	25.61891	24.37921	25.24296	25.93674	23.58589
P29622	31.05982	31.687	32.31041	31.68729	31.7862	31.74711	31.90387
P62805	23.50519		23.63079	25.52662		28.37538	
Q9UNN8	23.09603			23.89791		22.79788	
A0A087WSX0;A0A0G2JS06				25.17078	26.66601	25.1312	
Q92954	32.41906	33.92619	34.27482	33.44406	33.3645	33.1055	34.03919
P11047	26.39187		24.09297	25.8435	23.72411	24.24373	
Q6FHJ7			24.98681	24.36159	25.06295		
P26599	21.28218	24.19438	23.94485	23.98137	25.96655	24.39467	
O95445	26.86361	25.82583				25.64856	25.7102
P00338;Q6ZMR3;P07	24.92062	27.3157	25.90399	26.54311	27.73195	25.15054	26.45515
A1L4H1				25.79044		25.59656	26.21987
A0A075B6S5	27.18461	26.16317	26.32541	24.4837	25.80144	25.67274	26.14839

Q9UGM5		23.75195	26.01676	24.42419	24.81519	24.09515	
P02787	32.19269	32.47791	34.20354	32.57768	31.80116	31.979	32.51449
P20851	25.50679	25.55701		26.73982	25.07406	25.90617	25.67651
P06310;A0A075B6S6	29.93705	29.22789	29.27866	27.12603	28.73341	28.1883	29.42584
Q4LDE5	29.68674	30.13541	30.11272	30.20455	29.60555	30.46168	29.81432
P49747	27.15933	28.30943	27.87402	28.76696	27.96008	28.14846	28.80375
P05160;CON Q2TBQ	26.84577		24.50428	26.44727	25.38775	24.98637	25.15457
P01701	29.32879	28.90598	29.36774	28.28784	28.36018	27.59092	
P01714		28.48135	28.30239	26.4945	27.21419		27.63575
Q9UBP4	27.83539	25.7617		27.00378	27.28472	26.82202	27.68859
O43866	26.98826	26.98348	27.11138	26.24681	27.46762	27.1054	28.35909
P06396;REV Q6TDU	35.04629	35.54458	35.69654	34.98235	35.28926	35.14304	36.04757
A0A0C4DH68	28.86251	28.77036	28.84855	28.0488	28.43205	27.34376	27.5037
P02745	30.00625	28.43972	27.75506	29.80921	28.48439	29.25507	27.77121
A0A075B6K5;A0A075	28.45576	27.9511	27.87572	27.19272	28.74301	28.16581	29.32633
P02679	35.59463	34.40318	33.43253	34.99302	34.44924	34.75329	31.2917
P22352	24.60292		25.12353		23.97736	24.40944	
P68871	30.1321	31.70066	32.47588	28.4837	30.38597	30.11607	30.82206
Q14520	32.24969	30.12485	29.71117	30.48126	30.64453	30.67892	30.96623
P22792	27.9741	26.14299	26.31096	26.53754	26.30609	26.0793	26.74098
P08603	36.0028	35.41286	35.40882	35.77097	35.43313	35.52837	35.99108
Q3SYC2	26.93989	27.35473	29.03625	27.96025		28.54355	29.02918
P09211		23.33306	24.33142		24.90086		23.70841
Q99878;Q96KK5;Q9B	24.895	22.3093	24.96424	25.95022	22.18519	29.156	23.93148
A0M8Q6	26.40536	25.87124	25.79184		26.40547	26.7424	24.45901
P15311	26.94559	30.3198	28.94578	29.00453	30.74767	28.38365	29.39245
Q08431	25.41164	24.67239		25.83137		24.72739	
P0DMV9;P0DMV8;P3	23.3982	25.79117	23.6936	24.64361	25.39326	25.40441	24.71429
P01042;CON P0104	34.07264	33.3851	33.64817	33.6631	33.51981	33.11087	33.72069
P24043	25.60241		24.66671	25.06303	24.39709	24.42317	
P04003;CON Q2806	30.54729	30.11657	28.62062	32.19064	28.84182	31.19701	30.82145
P02675	35.6784	34.32482	33.40197	34.90015	34.09105	34.77676	31.02795
P98160	28.36064	28.64337	28.70659	28.95793	28.69737	28.99534	29.25142
P35542	28.83005		26.57074			27.37177	25.91796
Q96PD5;CON ENSE	31.07553	31.03394	31.08525	31.14291	31.27147	31.43788	30.88336
P02749	32.83812	33.90078	34.38516	32.95539	33.77676	32.70473	33.9775
P12109	24.59906	24.537	25.67066	25.75575	25.3812		24.28341
P07384	23.29221	25.02348	24.40724	24.37099	26.71371	24.78202	24.1055
Q5T749	24.58233	26.6392	24.13915		24.68816	24.43879	24.69163
P98095	26.80845	24.41629	25.20165	26.87443	25.05838	26.29763	
Q14767	25.04789	24.74484	24.6392		24.31458		24.81034
A0A0B4J1U7	26.93854		27.99583	27.60045	27.83485	26.92456	26.40866
P19823	33.03517	29.719	30.88336	30.67455	30.50673	30.54646	30.80977
P02790	32.31546	33.38317	32.556	32.81746	33.01365	32.99412	33.23948
P01344	25.21723		26.05902	24.11044	24.97998		26.16856
Q08380	31.69963	28.74076	28.3808	31.84599	27.69916	30.05634	27.69505
Q14574	23.62567	25.301	25.96023	24.72515	24.96556	25.02648	25.6818
P01619	30.05077	29.85975	30.45356	29.3204	29.66489	29.72038	30.68202
P00740	25.58362	24.44405	25.07427	24.30807	25.72985	25.2881	24.71623
Q92743	25.8327	26.46571	27.22564	25.80676	26.23854	25.88001	27.2048

A0A0A0MRZ8;P0443	28.16634	28.25296	29.48158	27.67681	27.68752	28.3966	30.28593
P26038	26.64485	28.72735	28.28016	27.94028	29.00584	28.4253	28.70273
P10643;Q12884	33.49774	32.61441	31.15412	33.61807	32.42997	33.32884	31.98043
P0DOY2;P0CF74	33.53589	33.77637	33.39892	32.47535	33.24799	33.17755	34.24629
A0A0C4DH38;A0A0J9YXX1		25.91757	26.59577		26.71293	25.82852	25.67441
A0A075B6K0	25.92028		26.95305			25.26152	
P05154	30.05956	29.87078	31.21899	30.22331	30.90734	30.00932	30.65275
P05155;CON P5044	34.50746	33.48939	33.59602	33.93921	33.61138	34.05415	34.15263
P60660;P14649		23.98997	24.34116	24.5618	25.76132	24.88502	
P04406;O14556	24.8388	28.62847	27.91211	27.49259	29.36986	28.00045	27.77838
P01871	30.49037	33.84978	32.15037	30.92635	31.67337	31.76117	33.38201
P07998		27.87619		26.12469	26.63104	26.641	
Q16270	26.37763	26.42119	27.57058	26.073	26.59662	26.03213	28.0071
P02743	31.12553	30.09497	28.39579	30.44184	29.04336	30.00021	29.85493
O00299	25.27785	25.07798	25.13199	25.72463	26.68258	24.84245	
P55058	28.61284	27.78544	29.70999	27.83581	27.94341	28.80427	28.24259
A0A0A0MS15		28.33424	27.12485	26.441	27.65935	26.49846	
P04114;CON	31.49329	32.75083	31.91449	33.61993	31.95522	34.28334	32.34797
P49908	30.14187	30.05336	30.03918	29.35328	30.12287	29.29362	29.57134
P02452	25.37132	25.50773	25.09136	25.46722		26.03635	
P69905;P02008	28.63502	30.34032	31.57799	27.32723	29.83568	28.98596	29.82164
P02766	29.03826	29.13293	30.12756	29.4435	29.76072	29.45727	28.38653
P34096		26.85875	26.17409	25.21985	25.96356	26.15329	25.64295
P06727;CON Q32PJ	31.6237	32.1461	32.08344	31.56225	31.32775	30.72712	31.10755
Q9H4M9	23.07798	26.36341	25.27483	24.92311	26.70464	24.24518	24.84609
P01009;P20848	33.23549	32.15912	32.15542	32.69054	31.67316	32.12278	31.68378
P04004;CON Q3ZBS	33.45366	32.54273	32.48145	32.73485	32.99429	33.04525	33.32174
P08571	26.95959	27.64231	29.25169	26.68739	28.06384	27.0626	27.63402
P05452	30.70767	31.02907	30.49686	29.21085	30.07227	30.2992	30.8674
Q12905		23.32526			24.93409	23.91743	
P02753	25.889	27.17064	27.88122	26.369	26.78544	26.2518	26.41658
A0A0C4DH73;P01611	25.18287	24.75583		23.05078	25.44297	24.21141	24.79828
P01024	37.11313	35.72069	36.24833	36.83552	36.23661	36.65611	36.37232
P13671	32.83096	31.67728	30.91371	32.45618	32.19072	32.3412	31.49004
P02647	34.07846	33.52648	34.24126	33.34876	33.48484	32.79054	33.92229
P59998				23.70081	24.47587	25.61723	
Q9Y6R7	25.00432	24.70462		25.29141	25.91559	24.54916	25.38037
P62937		26.18992	25.72907	25.21945	25.48987	25.24042	24.5952
P51884;CON Q0544	28.84852	28.91077	28.99064	28.36528	28.22619	28.79194	28.79597
Q99880;Q99879;Q99	24.25724	24.17767	25.3191	24.32334		28.38574	24.81729
P00325;P07327		27.75767	27.12928	25.74735	29.22777	24.98507	26.79922
P17936	28.61607	26.55123	26.47228	24.66443	28.18881	27.36545	27.25773
P27169	31.08759	29.10966	29.06879	29.88908	29.7614	29.12746	28.95989
P16035	23.91452	25.27526	25.10969		24.5661	24.68378	25.19749
P09429;B2RPK0	24.42515	25.58981	25.59155	25.5834	27.2249	25.00179	25.75404
P07585		24.18918	24.55544	25.58549	25.18219	25.25789	24.67352
P08697;CON P2880	30.2135	29.23619	30.1689	29.32745	29.75991	29.26754	29.46692
P43652	26.40279	25.47451	27.1211	26.34172	26.70767	25.71211	25.09378
P01834	34.81173	35.03838	34.2587	33.16975	33.79013	33.86073	35.60402
P01859	31.22717	32.83665	32.43654	31.02307	31.65442	32.22613	31.75971

P19827;CON Q0VC	31.20391	28.32376	28.94282	29.68001	29.0972	29.3709	29.1389
P05109	25.35619	25.78407	25.04864	25.358	25.53982	26.47376	24.19565
P39060	28.13306	28.08228	28.18428	27.35909	27.37119	28.11659	28.92612
Q14103		25.43106	25.04256	24.93989	26.51364	25.12692	
P24593	26.88122	27.08548	26.58291	25.45867	26.47605	25.67659	26.72806
O94985	24.86538	24.93256	26.03944	25.4617	25.44275	25.61871	26.07506
P62906		24.36766	25.0889	23.77826	25.53038	24.1715	
Q9NQ79	26.04816	26.62422	26.93674	27.12859	26.72689	25.78639	26.56426
P06702	23.98633	24.67428	24.07847		25.95031	25.46939	
P19021	27.61702	27.41256	26.53868	27.59562	27.99437	27.59861	28.49564
Q16610	30.63755	30.44766	30.15448	31.23746	30.9428	30.8589	30.05038
P19338		25.1189	24.95522	24.8189	27.6756	25.0618	25.05581
P04430	27.30391	24.77193	23.65611	23.735	25.25602	25.34357	25.59192
P02746	30.93481	29.68094	29.10505	30.99934	29.42976	30.70965	30.03
Q03591	32.2507	31.30286	31.5672	30.22561	30.37689	30.27186	32.53968
P11597	25.5308		26.77228	24.55684	24.73371	24.20568	
A0A075B6P5;A0A087	28.85267	28.44865	27.72312	27.30652	27.26123	27.21947	28.57286
Q7Z7G0		26.64485	27.35128	26.04293	26.06295	25.56909	25.86726
O14791	27.2507	26.67155	26.54022	26.16686	27.0041	26.27097	26.85828
P05156	28.98378	30.46285	31.49118	30.21917	31.42587	31.07903	31.33149
A0A0C4DH67;A0A0C4DH69			27.48784		25.4061		
P23381	23.36866	23.52456			22.97407	22.77394	
P18428	30.94014	30.58638	30.32892	30.30095	29.47042	30.47121	30.70446
P02652	26.61961		26.82214	25.6672	26.1212	27.27861	26.90644
P01034	26.91468	27.62645	27.70576	27.39713	27.41401	27.00721	26.41917
P24592	25.57479	27.20955	27.08507	26.06005	27.37997	25.97526	26.46888
P02788		23.46214	24.71046		23.14241	25.56317	25.05047
P09871	31.19272	29.75164	29.37387	30.89764	29.36062	30.19531	29.48232
Q9ULV4		25.70617	25.64738	24.70419	26.35949	25.71874	
P01023;P20742;CON_	28.56467	31.85259	32.03423	32.209	32.22605	30.99388	31.96973
Q01518;P40123	22.57711	26.94938	25.11132	25.64801	27.3379	27.07459	25.14363
P62249				23.86816	24.73091	24.47476	24.43275
P02774;CON Q3MH	30.26718	31.34223	32.62355	30.38072	31.35762	30.78613	31.71145
P36955;CON Q9512	33.24303	34.39036	33.75623	33.32335	33.9548	33.52063	34.31655
P00736	30.77148	30.21732	29.99739	31.12349	29.89319	30.53241	30.51991
Q9UK55;CON ENSE	27.25647	25.88821	26.39723	25.75995	26.30271	26.30231	26.99496
Q04756;CON ENSE	28.46556	27.85834	28.03023	27.95344	27.89903	27.5591	27.96069
P01861	28.37161	31.02426	31.7235	29.66892	30.82752	28.93944	30.52589
A0A0B4J1Y8	24.86575	24.90633	25.76058		24.79882	25.3503	25.12392
Q8WWA0;Q8WWU7	28.04422	29.39006	28.62031	29.11188	28.64413	29.21341	27.02538
P10909	31.30769	29.76191	31.05976	30.10462	30.87386	30.21419	30.21836
O75636		25.59335		25.07431	25.04935	25.32027	
P02649;CON Q0324	30.52636	29.33473	29.19324	30.23843	29.51614	30.50389	29.06702
P20774	23.91086	26.75226	27.52612	26.30847	27.57311	26.08703	26.30266
P00747;CON P0686	35.3176	33.63731	33.12923	34.83036	34.47677	34.77848	34.08654
P01019	27.54606	28.62756	28.39697	28.17279	27.07898	27.01287	27.72442
P02656	28.07444	28.47626	27.84978	27.56856	27.94559	27.80747	27.32697
P04075;P09972	26.30621	27.52671	26.58362	26.26578	27.98972	26.98869	26.54774
P03952;P20718	31.75282	31.76647	31.96534	31.51738	32.23164	31.26517	31.87504
P01031;CON Q1A7A	34.39917	32.51848	32.05433	34.12031	33.14625	33.91652	33.17705

P29401	22.85514	23.70208			25.82313	26.02827	23.93184
P07910;P0DMR1;O60	23.14158	24.79352	24.22986	24.14546	26.82202	26.14965	24.58302
Q13421	27.87883	28.87464	29.34659	28.28313	28.79334	27.93078	29.66536
P24821	24.42924	24.58612	24.88362	25.51045	23.82968	24.85057	
P01772;P0DP02;A0A0	26.48373	26.15516	27.67761	27.10219	24.82546		27.32971
P61978		25.46955	24.71466	24.93062	27.09848	25.3661	24.40282
P00751	34.10766	34.11062	34.06936	34.98363	33.88039	34.87381	34.20929
P51888			23.85671	23.32745	23.87736	23.1018	
P61626	29.17791	29.69969	30.11731	29.04001	29.36601	30.01425	28.67583
P00558;P07205		24.849	24.82377	23.96402	25.04485	24.32492	
P05546;CON	30.62601	29.31331	30.74855	30.25237	30.08487	29.56866	30.02327
P23083		26.98522	28.5458	25.81719	26.68966		
Q9BXR6	26.58047	24.92039	25.52016	26.45894	25.23612	25.46258	25.33323
Q15113	28.9632	29.24418	29.8378	27.85293	28.64141	28.3672	28.7457
O00468		23.77425	24.20165	24.18639			
P01857	36.19504	35.97999	36.2152	35.55751	35.84095	35.56618	36.26429
P02545	24.81744	26.48742	26.96433	27.14278	28.47317	28.82711	27.7239
P30041	24.34956	26.80218	27.01319	24.64053	27.80913	24.70382	26.90644
P04217;CON Q2KJF	29.47763	29.28848	30.09283	29.86331	29.1771	29.95615	30.07597
P00450	31.58915	30.58002	32.01069	31.0344	31.40177	31.00945	30.66907
P62987;P62979;P0CG	23.39976	24.04477		23.6767	24.10109	25.04172	
P12268		23.57126	23.64438	24.10813	25.01001	23.45664	23.77082
Q13822				23.49607	25.45804	23.84805	24.47004
P00739	26.81397	26.18092	26.44883	26.59249	25.09832	25.9331	26.55522
Q15149;P58107				24.22509	23.39558	25.28443	23.33634
P62081	24.65283	25.30124	23.57068	23.46076	24.02098	24.90325	24.06899
Q9HCB6			25.54407	23.45739	24.82886	24.52307	
P01880	32.11883	26.96587	28.06389	25.12206	28.86815	23.16154	
P80108			23.74499	23.66752	22.94106	23.54333	
P26927;Q2TV78	30.19589	29.77645	29.2595	30.14844	29.85448	29.87468	30.13259
P08294			25.73441		25.02855	24.0878	24.07096
O95833		24.75445			24.85262		
P31943;P55795;P5259	7	24.01563			24.50136	23.49863	23.69136
P07942	25.20311			24.46799		23.96464	
Q07960				23.39257	24.21542	23.29978	
Q8N1G4		23.83779		23.59263	24.80863	23.24156	23.36733
Q86UD1	24.08123		24.18124		24.16929		
P60174	23.09587			22.4788		23.40145	
P29350		23.41545			23.66134	23.33497	
P17931		24.07006	23.84105	23.87923	24.26971		24.51051
Q13838;O00148		23.53327			24.92502	23.49924	
P27105				24.57219	24.21052	24.61172	
P12956				25.29883	25.91882	24.01657	
P03973		25.05105	25.75799	25.30928	24.60925	25.60281	25.50461
Q06830;Q13162		24.89652		24.46837	24.94508	24.68806	24.08805
P13010		24.729		24.21771	24.81328	23.58806	
P04278			25.37488		23.65261		
P00742			24.4518	23.82192	24.40106	24.05656	
P62701;P22090;Q8TD47		23.36359	21.90342	22.78369	23.21414	23.32292	23.05353
P19971	23.33265				23.00879	22.68402	23.29249

O94903	23.15074	23.42445			24.19716	22.60304	
Q9NR45		23.348			25.67874	23.92171	
P30153;P30154			23.78935		23.66025	22.79442	23.25638
P11586		24.07586	24.47339		25.16592	24.22325	23.98494
P26641		24.48425	24.41551	24.28093	26.1142	25.23513	
P52907;P47755		23.66839	23.55415		24.17356	24.68421	
P37802		23.80971		22.96807	23.77626	23.1904	
P78371				23.03713	23.35891		
P61158;Q9C0K3;Q9P1U1		24.01461	23.93562	22.81982	24.32834	25.30469	
Q14195		24.69216		23.92624	25.90373		24.09362
P05388;Q8NHW5		22.89545		23.30243	24.80705	23.68378	
Q14204		24.30348	23.29628	24.30883	25.22986	23.99369	
Q15365		24.37232	25.31469	24.1519	26.61386	24.81982	
P22735				24.32231	24.18828	24.10229	
Q9H223		24.55789	24.02977		24.35696	24.72531	23.79432
Q12906		25.31969	24.1043		25.75453	24.46295	
P04899;P11488;P63096;P19087;		24.56517		24.46775	24.61156	24.08926	
Q86VP6			23.42816	23.32663	24.69434		
Q9NZN4			23.28404	23.70578	25.08282		24.18306
P35580		23.28771		24.98277	24.77415		
P49591		23.85529			24.57629	23.56302	
Q13642		25.8463	24.32971	24.71067	27.08487		
P49368		24.12408		23.76537	25.03418	23.92886	
Q15019	23.15328	24.36379	23.626	24.71093	24.83422	24.31934	
Q9Y3Z3	22.67088	24.00513	21.91707	23.19747	23.41918	27.42326	22.33006
A0A075B6R2			24.39414	24.93638			26.40267
O60749		24.75756			25.96774	25.80403	23.75011
P01877			25.30703	24.54097	24.71167		
P04632		24.97898		23.73985	25.78214	23.75532	24.7433
Q71U36;Q13748;Q6PEY2		24.65267			24.03792	22.72397	
Q15366;P57721		22.63533			24.40269	22.82394	
P23246		23.35542			24.32876		
P16403;P10412;P164	24.33456	25.06452	24.36038	25.17889	24.54286	26.76648	24.03078
Q01082			23.95934	24.68603	25.43838		
P26640		24.23717		23.84038	25.97871	23.02575	
O00232				26.04931	25.06213	25.60942	
Q06323		22.7946			23.46039		
O14745		23.95828	23.04955	24.76431	25.27213		
P46777		23.37994			24.09055	23.49228	23.634
O43707;Q08043;P35609;Q9H25		23.58738	22.6584	24.58354	24.38495	22.74382	
P11413		23.05094			24.07733	23.44063	
P50991		23.24729		23.11052	24.41067		
P0DJI8	25.2932	25.07312	23.26999		23.12293	26.77944	22.31072
O60506				23.26241	25.91543	24.15167	24.48573
P43490		24.27234			23.69763	24.53433	
Q9BXN1				23.72005	23.96763	24.45695	23.42547
P23396		22.67062	23.70282	23.48025	23.80006	23.43071	23.12025
Q6IBS0		23.21851			24.29158	24.26757	
P0DMN0;P0DMM9		24.68987			26.19672	25.40639	
P39019		24.6142	23.96084	24.78631	25.50909	24.3143	

Q9UQ80				23.65567	24.81		23.38179
P21810			22.62245	23.44594			
P63244		23.38311		23.55274	25.45522	24.06431	
Q9H8S9;Q7L9L4		22.67034			23.15411	23.25566	22.41949
P40227		23.5438			23.71911	23.23112	
P17858		23.52468	23.77706			24.17242	
Q15691		24.29397			24.85852	24.19686	24.85034
P07741		24.00213	23.74888	23.39637	24.32704	22.70369	
Q9Y678;Q9UBF2		23.5316		24.13101	23.94833	23.10072	
P15924	24.31783	27.68184	24.77916		24.17934	25.52573	
Q9UBX5	22.81531			25.63973	23.57323	25.08387	
O43776		24.47141			24.95677	24.33197	
P02750			23.10701	22.76191			
P50990				23.15905	23.56605		22.50809
P32119			26.78257	23.62411	23.97465		24.49869
P55072				25.42736	22.99118	23.54781	
P51812		23.42355	23.34272		24.61054		
Q96S96		25.25256	25.31772	24.44929	24.16315		
P28799						23.22719	
Q92841					24.81284	24.47593	
P09382					23.45576	23.37677	
Q93091		23.60817				23.55485	
P35268					24.46314	23.74612	
O75083		23.27441				23.24657	
Q10567					23.82211	23.85024	
Q14152				23.29081			
P50225;P50226					25.12222		
P46781				23.80941		23.94735	
P62269					23.43873	24.3152	
P84243;Q71DI3;Q166	26.11527					27.78188	
P43686		23.0219				23.33402	23.44935
Q9UL46					23.41403		
O14773		23.71984				24.8004	
P29692				23.63755		23.8812	
Q6UXB8			23.55028				
Q8NHP1;O43488;O95154		23.05078					
A0A075B6I0	25.3675						
A0A0A0MT36					26.1727		
A0A0B4J1V6							
A0A0B4J1X8							
A0A0B4J1Y9					26.10998		
A0A0C4DH24					23.73614		
A0A0C4DH29			22.45141				
A0A0C4DH34			24.45356				
A0A0J9YX35					23.4763		
P14598;A6NI72;A8MVU1		23.36145			24.00187	24.71602	
A8MT33					26.90724		
Q99613;B5ME19							
Q13765;E9PAV3;Q9BZK3							
O00151					24.10629		

O00159					22.82324	
O00160						
O00182;Q6DKI2;Q3B8N2						21.32619
O00231					24.93674	
O00303						
O00571;O15523					24.1292	
O14498						
O14979		24.18495				
O15078						
O15143						24.62064
O43240						
O43390						
O43813					23.58692	
O60493						
O60701		23.53825			24.32999	
O60763						
O60826						
O75339	23.91843			20.79574		
O75367						23.53837
O75390			22.55309			
O75489						
O75608						
O75828						
O94804						23.61829
O94973					24.28306	23.47457
O94979						
O94991						
O95050						
O95336						
O95340		24.092				
O95433					21.22406	
O95466						
O95479						23.65513
P00326						
P00966		28.11585		28.05843	28.22715	
P01594;P01593					26.32524	
P01601					23.69785	
P01703					24.95314	
P01717		23.96473				
P01766					24.97985	
P0DP03;P01768;P01764;A0A075B6Q5					26.8147	
P02511		23.47766			23.16082	
P02549						
P02655						23.18374
P02730	22.19958	22.67135				
P02741						26.33388
P02775						
P02792						25.12723
P04070						
P04179						22.50807

P04180	24.1118						
P04843							
P04844							
P05062					22.33911		
P05091;P47895;P30837;O94788						24.49131	
P05107						23.42432	
P05141;Q9H0C2						24.15167	
P05198					23.32237		
P05976							
P06576	23.826	23.44342		23.81		25.34878	
P06737;P11217;P11216							
P07203							
P07237						24.32444	
P07305						23.69275	
P07737							
P07814							
P08237							
P08493							25.20624
P08567						23.17106	
P08582							
P08865							
P09467					23.78367		
P09486	24.1425			24.01979			
P09525				22.79237			
P09651;Q32P51							
P09874							
P0DJI9							
P0DP01		28.35758	27.60357				
P0DP04			27.38542				
P0DP25;P0DP24;P0DP23							
P0DPA2						25.84561	
P10809						23.9104	
P11277							
P11279						23.94387	
P60891;P21108;P11908			23.56941				
P12429				24.62979	24.65671		
P12814							
P13489							
P13591							
P13667						21.14046	
P13716							
P14317							
P14866							
P14868							
P15144							
P15153;P63000;P60763							
P15259;P18669;Q8N0Y7							
P15814					24.6355		
P15880						22.7074	
P16050		24.45494	24.96001		24.59673		

P16157						
P16401						24.82386
P16452						
P16870			24.08335			
P17066;P48741					23.77756	
P17900						
P17987						
P18124						
P18206						
P20039;Q9TQE0;Q30134;Q29974;P79483;P13761;P04229;Q95IE3;Q5Y7A7;P						24.1495
P20591;P20592		23.62422				23.49106
P20700						24.27327
P21266;P46439;P28161						
P21291						
P21399				21.67224	23.60298	
P21817	25.52232			24.43454		
P21980					23.13558	
P22061					23.20701	
P22234						
P22314						
P22392;P15531				23.6725		
P23284						
P25398						
P25705						26.87959
P26368						
P26373						
P26583						
P27361;P31152;Q16659		24.94391			24.89888	
P27695	23.35488	23.08944		22.95977	25.63942	
P27797						24.87497
P27816					24.28327	
P27824						23.53777
P28838						23.17473
P29466						
P29508;P48594						23.03194
P29972						
P30043				24.41667		
P30048						22.86456
P30050					25.31029	
P31939					23.77957	
P31948		22.66505		22.47586		
P33176;O60282;Q12840						
P35232						
P35270		22.79188			23.06773	
P35606				22.87487	23.25566	22.83341
P35998						
P36543						
P38159;Q96E39;O75526;Q8N7X1					23.34773	
P38606						
P38919					21.54628	

P40121					23.85396		
P40261					24.68656		
P40763							
P40926						23.07946	
P40939							
Q2VIR3;P41091							
P41218						24.50809	
P41240						23.52981	
P42224		24.17942			24.08513	23.68849	
P43251				22.06177			
P45974							
P46459							
P46782							
P47989				22.57344			
P48426;P78356							
P48444							
P48643							
P48735						23.94136	
P48740	25.6064						
P49411						24.49051	
P49748							
P50454				23.70324	23.76486		
P50552					22.43104		
P50570;Q9UQ16							
P51148;P61020;P20339							
P51149							
P51570							
P51991							
P52209						24.25911	
P52565							
P53621							
P54136							
P54577							
P55036							
P55084						22.87008	
P55209;Q99733					21.04427		
P55884							
P59666;P59665						24.04402	
P60228							
P60866						23.18953	
P60903				23.78187		25.47698	
P61006;Q9H0U4;Q92928;P51153;P62820;Q92930;P59190							
P61160						25.15858	
P61163;P42025 P61247							
P61353							
P61769	25.13222						
P61981							24.78352
P62136;P36873;P62140 P62191							

P62195;Q8NB90							
P62241							
P62244						25.0089	
P62258				22.84454			
P62424						23.76243	
P62750							
P62851						24.41815	
P62873							
P62888						24.71749	
P62917				22.79735			
P68133;P68032;P63267;P62736							
P68371;P04350							24.90389
P69892;P69891							
P84095							
P84103							
Q02413						22.75252	
Q02878				22.74277		22.69886	
Q02985;Q92496			26.5513				
Q03252							
Q04826;P30488;P30487;P30485;P30483;P30481;P30461;P30479;P30480;P30460;P01889 Q04917							
Q05682		23.33197			23.75746		
Q08211					24.31852	23.67519	
Q08257					24.04743		
Q09666						24.06842	
Q0VF96							26.35326
Q13045					23.35016		
Q13103	24.61324						
Q13200					23.57842		
Q13228							
Q13263					22.61162		
Q13283							
Q13310		22.3431					
Q13332				23.55661			
Q13492;O60641						23.97413	
Q13596					21.15047		
Q13813					24.47352		
Q13885							
Q14203							
Q14258							
Q14315							
Q14697						24.03255	
Q14764						23.21727	
Q14974					22.0475		
Q15008					22.96506	22.79699	
Q15046							
Q15056							
Q15084						25.32382	24.51737
Q15435					21.67276		
Q15717;Q12926;P26378							

Q15813	25.15167				
Q15942			23.2415	23.89227	
Q16181					
Q16627		24.32265			
Q16647					
Q16658					
Q16666			22.89123		22.93047
Q53EL6			23.47209		
Q58FF8				21.99862	
Q8N2N9;Q5JPF3;A6QL64					
Q5SSJ5					
Q5SYB0			27.96955	28.92397	
Q6PIU2					20.9315
Q6UY14		23.88362			
Q7KZF4			24.13649		
Q7L576;Q96F07					
Q86UX7	23.83441	23.04873	22.37584	23.78616	25.4903
Q86VB7					25.46023
Q8N2S1				24.2169	
Q8N392					
Q8N474		25.82554			
Q8N6C8;O75019;Q8NHL6				24.70398	
Q8TAT6					
Q8TER0					
Q8WUM4	22.9996			23.69147	
Q8WYP5					
Q8WZ42		24.4737			
Q92499				23.9331	23.14736
Q92522					
Q92530					
Q92599					
Q92859			21.80093		
Q92900					
Q93052					
Q96AE4;Q92945				24.18835	
Q96AG4				22.62943	
Q96C23					
Q96CW1					
Q96CX2					
Q96H15					
Q96HC4	24.61628			24.53077	
Q96KP4					23.75654
Q96QK1				22.5426	
Q96T37					
Q99536				23.89421	
Q99538					
Q99784					23.40028
Q99829					
Q99832	24.73758				
Q9BQT9				23.13648	

Q9BUJ2		22.11607				
Q9H008					22.85702	
Q9HB71						
Q9HDC9						
Q9NSD9					23.22817	
Q9NTK5						
Q9NU22	26.2217					
Q9NUV9;Q6P9H5		24.55883				25.06755
Q9NVA2						
Q9NZA1;Q96NY7		24.59212	24.31996	24.32237	26.24207	
Q9P2S5						
Q9UBI6						
Q9UHD8						
Q9UNH7				23.27923	24.30452	24.8276
Q9UNW1						
Q9Y224					19.34715	
Q9Y262						
Q9Y265						21.54811
Q9Y2Q3						
Q9Y3I0					23.74776	
Q9Y5X3		23.79828				23.78287
Q9Y696					24.41319	
Q9Y6N5						

8	9	10	11	12
26.79736	24.70572	25.3098	24.71219	26.1536
	23.16687			23.06848
30.42802	27.06198	29.57431	28.56747	27.65041
27.69478	23.63589	24.52163	24.87961	25.89934
29.19357	26.64251	25.74332	25.70179	27.13438
28.46416	29.6939	28.63183	29.09489	28.54425
28.90613	27.03328	26.32603	27.73337	27.09899
24.73542		24.04285	23.99705	23.79392
29.25854	28.03007	26.88553	25.86441	29.43169
29.73101	30.04895	30.48068	29.6231	29.65799
28.72559	26.73001	26.57715	27.63513	28.17031
25.82202	24.4288	23.23661	23.80892	24.47822
25.10121	25.31268		25.37305	25.47417
24.87145	23.10575			23.53065
25.80035	24.45757	24.20561		26.02521
26.28419	23.84652	23.74242		26.4807
31.92956	30.54757	29.73804	30.3255	31.22043
25.7417	23.17501	23.66112	23.47333	26.08406
21.83418	23.25226		23.63567	22.54503
28.63696	24.81548	24.71125		28.32569
31.13216	29.61319	29.6481	30.30389	29.65164
26.9888	26.65267	26.19648	28.0248	25.35783
27.20769	26.82929	26.62826	26.89781	26.81421
26.00009	27.89914	28.06999	27.10139	26.37751
32.19096	32.36152	33.43502	32.83387	33.21489
29.55779	30.29033	29.50938	29.77211	30.15918
27.93955	28.05729	28.34227	28.48078	28.54348
32.75249	34.67235	33.76535	34.6493	34.66654
26.67061		26.03804	26.06686	25.9007
24.97443	24.8579	24.99274	24.56819	
32.95095	32.00608	31.5607	31.20915	31.39545
	25.94022		25.55476	24.61217
28.89553	28.87223	28.32053	28.60855	28.57939
27.33892	25.37179	22.99314		27.64526
		24.20583		24.08691
25.45001	24.75608		24.96482	23.51075
26.01772	23.6621	23.04926		24.69615
30.15967	27.45364	26.52637	25.79174	30.52832
26.83014	24.17798	25.27327	23.63367	26.47934
27.88483	27.72917	27.0748	26.89215	28.68833
26.66397	25.69445	24.11068	24.76961	24.79169
23.88911	23.16426	23.78885	23.43466	24.41886
31.87416	31.5819	31.04446	31.13357	31.33926
25.60767	22.96876			24.16338
26.93224	24.0312		23.36399	26.60428
27.99577	26.11188	24.36005	23.97439	25.12589
27.14316	25.61198	26.33294	27.01765	

24.50706	25.92329		24.81142	24.2203
25.61321	24.45745		23.12663	26.30847
25.55675	26.35525	25.78517	27.30609	27.33833
26.33301	26.28801	27.32997	25.82308	26.66329
29.03682	28.39157	27.83455	28.92717	28.54698
26.34006	28.92218	25.89684	26.98294	26.74676
29.28036	28.24984	27.55815	27.30095	29.68264
24.97561	20.88539			25.40175
	23.34665	23.83258		23.94271
31.22711	30.23261	30.14747	30.45914	29.4091
25.99423	25.74555	24.76512	24.16829	27.61084
28.67189	29.13592	28.71467	30.06689	28.20923
23.71366	23.08398	22.97411	22.93745	24.30918
22.30536	30.03905			25.80794
	25.19985	25.10177	24.62801	
27.17131	24.28355	22.0791		24.12471
	23.68614	23.47135	24.91205	25.44421
29.8998	30.50786	29.64387	30.02221	28.36711
27.50711	25.24067	24.4642	25.16982	27.54385
28.97145	27.08952	27.17265	25.82753	29.47402
30.47663	30.58271	31.28731	31.37322	31.71972
28.09381	27.54834	26.86254	26.96686	27.73751
27.97918	25.87436	25.9115	24.03271	26.71803
28.30104	28.06666	28.62024	27.68732	27.98163
28.47981	27.09828	25.15101	27.49937	27.07612
23.7416	22.79664			23.20896
31.12522	30.36974	31.51197	31.3453	31.75139
35.66055	35.72245	35.57142	34.96624	35.66066
25.49011	23.5064			24.74842
27.14472	26.89828	25.16726	25.82236	27.31086
25.32337	24.26914		24.08091	24.96349
25.8381	25.3677	25.13782	25.45249	24.60642
34.10821	34.71124	35.57816	35.17128	35.05051
31.61281	30.97624	31.16591	31.14613	28.41667
31.1543	30.7809	30.83583	30.50085	31.13241
25.5415	25.48938	23.67648	24.25645	23.50978
30.48558	30.50786	31.19795	30.95297	29.71311
27.98136	29.08418	28.29986	27.46817	28.63506
32.00558	32.38862	31.63218	32.40169	32.50368
27.02253	27.1035	27.43698	25.82437	26.5969
25.32968	24.48726	24.61324	25.00736	24.40536
29.74482	28.53501	28.83307	27.97962	28.87256
23.80065	23.27654	23.83961	23.60603	24.01453
	24.00385		24.35871	25.32406
23.80459	23.05489		23.27384	23.92098
33.66342	32.90598	33.18779	32.73113	32.57016
27.16125	26.79179	27.07663	27.23316	27.40227
24.4367	25.19708	24.4742	25.25588	
29.19039	25.91643	24.45413	23.54168	28.11981
	25.55315	26.05519	25.61175	

26.08928	26.06198	27.02105	26.29995	28.51277
24.4263		23.55555		25.26607
27.44166	27.50385	27.61224	27.6047	28.19943
24.3585		24.79585	23.17617	24.3891
32.59123	32.89901	31.87537	32.51496	32.46205
	27.46428			26.61049
26.86112	23.48763			27.33347
29.45717	28.65072	29.04856	29.6098	29.75049
33.94148	33.09027	34.22482	31.92061	33.37542
25.80496	25.84871	25.94045	26.01708	26.17108
28.59488	28.80169	28.5189	29.03674	28.30435
35.0333	34.07296	34.46958	34.03379	33.08065
31.68479	31.93758	31.83981	31.63487	32.85432
29.21039	28.73421	28.23114	26.99873	27.82274
30.74535	29.87569	30.22826	30.86968	31.53538
27.9173	23.4148	24.37557		24.80877
26.35963	25.74635	25.57479	25.6174	28.0402
24.5125	24.67266	25.10518	24.52115	24.75588
24.7959	24.58009	23.94913	23.90573	
		23.26999	24.87098	24.16438
31.20536	31.8633	31.22452	30.85423	29.88956
29.50658	30.4006	30.96265	30.72955	31.60245
26.31736	27.23936	26.28171	27.07592	25.55353
25.66232	24.50082	22.86028	24.39611	25.63603
	23.29025		23.1284	
23.87202		23.51713	23.66525	
			24.37643	24.07243
32.81004	33.27434	33.96189	33.04037	33.52892
25.90644		22.76715		26.19558
26.77731	24.83137	25.11815	26.93753	27.35313
27.17817	25.71542	24.17752	25.1565	
30.24615	30.78542	32.4035	31.48039	31.25417
	24.55274		23.89199	
	24.32739		23.05654	
29.73552	29.58508	29.55571	29.57643	30.73182
24.20322	25.78144	23.69551	25.58557	23.02459
29.00587	29.80775	29.74034	29.71782	29.84047
24.34564	24.66844	23.62411	23.57957	25.60764
31.3818	31.82574	32.10759	31.70722	31.86308
			23.03726	24.04968
25.21974	23.14737		23.10546	23.15579
	25.58451		24.6036	25.07549
32.95114	33.68781	33.34109	33.35061	34.78166
25.29313	24.28496	25.30198	23.17604	
			25.29509	24.93179
24.28546		24.00033		26.00415
	25.79385	25.79986	25.49823	
28.05641	26.28873	24.76405	25.32413	27.25997
	25.4932	25.71539	25.38281	26.04968
24.86849	26.44234	26.08182	25.74258	26.61161

23.65042			24.39924	25.80373
32.28601	30.59895	32.63324	31.20005	33.57835
26.80796	26.87666	25.43017	26.57802	
28.67381	28.72306	28.35267	28.64227	28.04661
29.63836	30.02049	30.36223	29.64638	30.48587
28.17164	28.15224	28.08836	28.48347	26.61189
26.67694	25.40344	25.53893	25.09229	25.25947
27.97547		27.58197	27.68679	29.12925
		27.47568	26.88274	27.28047
25.61493	26.20174	26.34177	26.75468	
29.07646	28.28348	27.00174	28.11937	27.81647
34.59301	35.41972	35.44758	35.41963	35.61774
28.58474	28.46248	28.83491	28.48945	28.38718
29.87872	29.71276	29.20119	29.86106	28.51081
29.16327	29.83584	27.7442	28.71503	28.55435
34.99648	33.84903	34.22425	33.76821	32.9632
		24.56483		25.12715
29.49242	35.24619	30.14127	27.37327	33.00576
30.25946	30.68879	30.91784	30.07329	29.55461
26.33098	26.26051	26.78531	26.15252	26.01144
35.24307	35.67311	35.66715	35.79711	35.70301
	28.30326	26.54569	28.40771	27.62275
		22.74224		24.29334
21.12168	22.20222	21.88837	22.46665	26.34035
25.329	24.48401	24.9428	27.25872	24.97382
29.39317	28.68786	28.05072	27.6013	30.16207
26.51301	24.80813	25.45174	24.94806	
24.85191	24.76714	23.021	24.55157	25.07255
33.30144	33.16722	33.71927	33.60521	33.20915
24.60422	24.56302	24.89398	24.26942	
32.02782	32.26665	31.84229	32.26858	28.40479
34.84066	33.59424	34.15202	33.88039	32.84846
27.95887	28.92852	29.71168	28.75242	28.1891
27.26132	27.39558	26.51039	26.60329	26.66777
30.72964	31.95837	31.32678	31.69669	30.1321
33.42229	33.2453	34.00872	34.00696	33.61927
25.26467	24.76481	25.06936	24.62684	25.0065
24.85719	24.58658	22.70807	24.35447	25.49039
	24.33763		24.30855	24.87037
24.17714		25.35313		24.67551
24.50615		24.4711	24.63849	
28.00088		26.36386	26.3945	28.38755
29.36734	30.40152	30.5505	30.23055	30.23763
33.5632	32.21292	33.16796	33.45145	32.40454
		26.26821	25.25079	25.42082
30.55783	30.77211	30.74631	30.91584	28.20611
23.45626	24.89194	24.951	24.29193	26.55314
30.50607	29.5456	29.79957	30.21836	29.96155
25.24147	25.43212		24.37126	24.7305
26.21791	26.23037	26.43124	26.37743	27.13448

28.25273	28.08542		28.66465	29.86865
28.19826	28.35762	26.98685	28.00131	28.623
34.0006	33.02535	33.0672	33.18105	30.59584
32.87827	33.67766	33.39036	32.89777	32.70207
27.22371		27.75557	25.14383	27.13497
26.94067	25.79877		26.30509	25.83764
30.02433	29.6759	30.97261	30.60001	30.94356
34.36365	34.4855	34.47635	33.97254	33.46283
24.26542	24.07071	23.64328		25.36866
28.64231	27.48255	26.57454	26.66763	28.94578
33.76159	33.46794	31.48951	32.79807	32.74677
27.12682	27.44963	26.42321	25.94568	
25.93096	26.73027	27.71221	26.13316	26.86396
30.79452	29.9735	30.98685	29.45452	28.21586
26.83316		24.39382		26.16988
28.60445	29.82246	27.26177	29.93466	27.85061
26.99949	25.87162	26.82529	27.23535	27.81232
33.80761	33.70378	33.6954	33.40146	31.5037
29.28103	29.64964	29.69616	29.98373	30.00544
25.88246	25.62492	25.3506	26.32759	25.41832
28.47448	34.10095	28.83846	25.76142	31.40487
28.57726	28.95238	29.71268	28.76199	30.23706
25.14204	25.39718	25.28803	26.10183	26.08503
31.01585	31.48534	30.4183	31.56565	31.72749
25.83357	23.37716	22.65147		27.86997
32.29939	32.40007	31.44174	32.5009	32.1029
32.29956	32.8393	33.30959	32.08901	32.7094
26.83497	27.18848	27.03328	27.51818	29.33994
29.50531	30.06368	30.56311	30.20478	30.90038
23.43441	23.63677			24.78077
26.33686	25.60733	26.72754	26.00039	27.47112
24.18117	23.60433		24.13108	26.81066
36.29842	36.42444	36.77497	36.25917	35.86507
32.36328	32.32876	32.76123	31.86474	30.41448
32.97897	33.93807	33.07216	33.71211	33.94288
23.49936	23.71942			24.31237
27.40868	24.67368	24.15499	26.68177	
25.32509	25.06517	24.93423		25.79699
27.62324	28.55702	28.28167	28.11048	28.90991
23.92949			25.13108	25.81909
26.31884		26.09458		28.48405
26.58506	25.69479	28.48001	26.88075	25.65105
29.43265	29.92663	29.23165	29.65265	28.8165
24.315	24.15229	25.62915	23.96314	24.59594
26.02327		23.9534		26.75098
25.57254		24.80371		24.90862
28.80193	29.31896	29.30361	29.76745	30.12324
25.12143	25.77289	25.48216	26.19291	27.17446
33.94419	34.20456	34.2996	33.92389	34.25505
32.58233	31.93326	32.63921	31.06638	31.69611

28.5263	29.30643	29.69856	29.6634	29.09899
24.34618	25.65638	24.74888	24.71984	26.57168
27.92643	27.79489	27.94073	27.33816	28.56776
26.03133	23.61784			26.81311
26.00755	25.93847	26.77731	26.35165	26.81336
25.13081	25.70306	24.85648	24.55485	26.21963
24.74622			22.71565	25.95363
25.06225	26.85555	25.43657	26.59278	27.0663
	25.09708		24.13422	24.77545
26.34374	27.75729	28.28476	27.4235	26.81421
30.40385	30.52159	31.37979	30.76462	30.02168
25.62419		22.19698	25.89356	27.3955
25.33323	23.80006		25.61403	26.65362
31.24644	30.97596	30.66153	30.47557	29.47665
31.53895	31.60528	31.51597	30.90792	30.46402
24.35043			24.5291	25.11307
27.72071	28.29798	28.10904	27.58885	27.15045
25.35978	25.87432	25.60082	25.00002	27.49251
	27.52395	26.82663	25.97264	26.05986
30.90239	30.85556	30.85341	31.3156	30.37265
25.28253			25.57372	26.05063
	22.86003	22.39739		23.90518
30.40446	30.37233	30.1689	30.70792	30.58073
24.57109	26.90357	27.09336	25.32327	26.34645
26.66316	27.04937	26.66953	27.48155	27.64003
26.36815	26.32658	25.91762	27.022	27.1576
	24.78761		23.58302	
31.2198	30.76169	30.32517	30.5538	29.51554
25.13218	24.72676			26.65171
32.09427	31.79405	31.4828	31.63781	31.62714
25.48367	24.90546	24.29733		27.1043
		23.95607		24.98807
30.52412	30.77431	30.52244	30.40446	32.36048
33.73024	33.92158	33.79634	33.76643	33.81593
31.19372	31.07839	30.45425	30.90462	29.93185
25.82277	26.02574	27.47321	25.92581	26.2245
28.40864	28.15702	28.29772	27.81305	27.65963
27.81109	30.61105	28.83957	29.6717	29.76253
	25.34164	25.36493	25.09861	24.55731
28.66353	28.87288	28.87159	27.21799	29.77527
30.20233	30.73174	29.80962	29.95352	31.13742
26.04901	26.03882	25.25811	24.76643	
29.61537	30.49066	29.79497	29.98495	28.4479
26.44438	25.90382	26.38594	25.61153	27.9975
34.75901	34.54375	35.1479	34.82781	33.27281
27.25061	27.69312	27.05186	27.59612	28.34041
26.77128	28.60802	27.36903	26.78718	27.91843
27.5515	27.32852	26.73505	26.72403	26.88367
32.07368	31.53955	31.98325	31.87427	31.73113
33.95116	33.67219	33.79836	34.28057	31.56878

24.41654	24.0333	24.42758	24.90894	24.14336
24.9983	23.46712			26.0023
28.86576	27.51991	28.01425	27.85091	31.12195
25.24616	24.89152	24.66487	24.84173	
26.02656		25.4303	27.29088	27.04646
26.26322	24.00213			26.72923
34.68275	34.38561	34.65256	34.67225	33.66023
	22.72124			24.26614
28.76072	29.39313	28.9979	28.90357	30.24139
25.18052			24.48388	24.96957
29.39946	29.60943	30.11744	30.11458	30.25744
24.8243	26.15943	28.22168	25.47212	26.70266
26.18331	25.72666	25.67128	25.18658	26.16287
28.52806	28.26114	28.01802	29.68662	29.23879
	23.61201	24.4431		
35.73711	35.82693	35.88704	36.03872	35.88999
25.49972	26.76699	27.239	25.61863	28.16269
26.87818	23.94869			28.46529
29.43754	29.80058	29.33739	29.60855	29.97104
31.35042	31.3452	31.10224	30.98848	31.42627
24.14632	24.17577	24.04068	23.95438	
24.87858		22.88796		25.28221
	25.17146	23.74273	24.53487	23.75542
28.20275	26.14293	26.55296	26.9149	26.3537
23.19963	22.96007		23.52779	25.12151
23.84728	23.81343			24.61958
			23.96314	25.17832
26.00622	26.24327	23.86098	29.96444	28.5953
22.61214	23.98442	23.41622	22.8225	22.98829
28.81928	29.68562	30.18471	29.1688	29.99456
23.70841				
24.32876				24.01274
24.50694			23.89938	
23.20304			23.16906	
23.39297				23.97342
24.09063				23.8258
23.41235				
				23.10226
23.81558				25.45811
22.87832				23.74755
25.03292				
25.48603				24.8924
	23.96965			
24.63179				
24.52815				
22.01964			23.92506	
				25.98748
	22.56558			
	24.41384			

23.14325				23.74139
23.63012				23.51002
23.9402				
24.77515				24.17219
25.18835				25.31603
23.67917				23.91123
				25.22946
23.18277				23.8839
23.09012	22.90367			
24.79927				24.19205
23.78646				25.39813
24.88925				25.23385
25.71947				26.42562
25.41509				
23.99092				24.00033
24.37312				25.21004
25.94753				
23.89901				24.57462
23.42765				26.63367
25.62564				24.21897
				24.19963
24.85262				24.96776
24.25739				26.14562
23.92597				25.01844
21.92071				23.17136
24.25566				
24.4367				25.81034
24.76137			25.30166	
24.87479				24.85081
24.08489				25.00577
24.24133				24.86108
23.85167				24.33885
23.46513				25.52567
24.44082				24.54528
24.61178				25.40354
25.87368				
22.75314				23.22105
25.43882				23.64966
24.18533				24.03523
25.21826				24.13891
23.37332				
23.90976				23.55344
	24.26342			
24.59474				26.02941
				23.6712
				23.89551
23.39989				23.94789
		23.51905		
24.3199				26.07668
25.06336				25.01359

24.09603				24.82876
23.27767		22.59544		
23.85377				24.17326
23.4821				24.05681
				23.22052
24.02411				25.3761
23.38126				25.68126
24.58698				23.99058
24.81083			25.29821	
23.96058			22.8939	
				25.11886
22.70071				23.17705
23.83567				23.76091
24.0059				25.90651
26.23705		22.81505		
23.59366				23.98459
24.06398		25.53184		
	23.24825	23.08123		
				24.56692
22.97474				23.8349
23.70599				23.98503
				24.17043
23.05743				23.43173
23.67681				
23.29586				24.25306
23.28658				24.87577
23.53124				
				23.28588
				24.95247
22.71047				24.76481
				24.4729
24.34293				24.61941
			23.0488	23.514
23.03342				24.59497
			26.24885	
21.62921				
23.98989				

22.74489

25.47948

24.29845

23.90343

24.15437

29.64904 26.70477

24.1187

26.86573

28.4453

25.20736

23.28334

23.0017 23.84431

26.91855 27.59049

24.90876

22.50797
22.80434

22.13013

23.67347
24.6615

23.30577
25.09897 24.59457
24.91921
24.06036 24.87375

22.17588

28.39133

23.87051
26.08582

23.89403

25.15167

23.85852

23.91488

22.34291

24.81973

23.07421

22.68648

26.39824

25.56387

25.44717

25.55734

22.77006

23.1957

21.39242

23.43479

25.06229
23.84957 24.44006

23.66232

23.43784 24.02985
23.37425

24.10845 23.62779

23.08047

24.74335

23.55098
23.91579
23.73076
22.38664
23.31396
22.88308
22.96885
24.25782
23.68859
24.89805

23.32841

24.60801

24.45023

23.07773

24.16415

22.25442

23.38469 23.61829

23.11027

24.28185

23.27597

22.84077

24.25983

24.92656 23.93391

24.12526

23.55812

23.45073

25.40497

23.47828 23.0729

23.47902

20.93855

22.21386

24.95522

25.30016

23.27569

22.26442

24.65551

24.81367

25.037
